# The Atg1–kinase complex tethers Atg9-vesicles to initiate autophagy

**DOI:** 10.1038/ncomms10338

**Published:** 2016-01-12

**Authors:** Yijian Rao, Marco G. Perna, Benjamin Hofmann, Viola Beier, Thomas Wollert

**Affiliations:** 1 Molecular Membrane and Organelle Biology, Max Planck Institute of Biochemistry, Am Klopferspitz 18, Martinsried 82152, Germany

## Abstract

Autophagosomes are double-membrane vesicles that sequester cytoplasmic material for lysosomal degradation. Their biogenesis is initiated by recruitment of Atg9-vesicles to the phagophore assembly site. This process depends on the regulated activation of the Atg1–kinase complex. However, the underlying molecular mechanism remains unclear. Here we reconstitute this early step in autophagy from purified components *in vitro*. We find that on assembly from its cytoplasmic subcomplexes, the Atg1–kinase complex becomes activated, enabling it to recruit and tether Atg9-vesicles. The scaffolding protein Atg17 targets the Atg1–kinase complex to autophagic membranes by specifically recognizing the membrane protein Atg9. This interaction is inhibited by the two regulatory subunits Atg31 and Atg29. Engagement of the Atg1–Atg13 subcomplex restores the Atg9-binding and membrane-tethering activity of Atg17. Our data help to unravel the mechanism that controls Atg17-mediated tethering of Atg9-vesicles, providing the molecular basis to understand initiation of autophagosome-biogenesis.

## Abstract (web summary)

Autophagosome biogenesis is initiated by recruitment of Atg9-vesicles to the phagophore assembly site. Here Rao *et al.* use a reconstituted *in vitro* system to describe the mechanism by which activation of the Atg1-kinase complex from its constituent parts recruits and tethers Atg9-vesicles.

## Graphical abstract

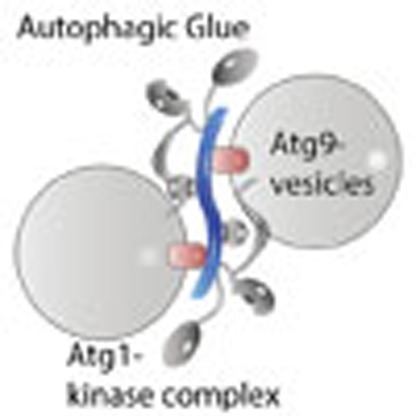

The homeostasis of cells depends critically on recycling pathways to remove superfluous or damaged cytoplasmic material^1^. During macroautophagy, to which we will refer to as autophagy in the following, cytoplasmic cargo is engulfed by a cup-shaped membrane, termed phagophore. Formation of such phagophores requires, in addition to canonical membrane remodelling and fusion machines, a dedicated set of autophagy-related (Atg) proteins^2^. Most Atg-proteins have first been identified in yeast^3^,^4^,^5^, but many of them possess mammalian homologues, emphasizing the high degree of conservation among eukaryotes^6^.

Autophagy is initiated at the phagophore assembly site (PAS) in yeast, to which Atg-proteins and donor-membranes are recruited in a spatiotemporally coordinated manner^7^,^8^. Sequestration of cargo from the cytoplasm involves expansion of the phagophore. Sealing of the membrane generates the double-membrane surrounded autophagosome. Enclosed autophagic cargo is finally delivered to the vacuole for degradation^9^.

Although Atg-proteins have been studied extensively^9^, a central question in autophagy research concerning the nucleation of autophagosomes remained unclear. A recent study suggests that an average of three Atg9-vesicles coalesce at the PAS^10^. The recruitment of these vesicles depends on the scaffolding protein Atg17, which is the earliest protein to arrive at the PAS^7^. Atg17 adapts a highly elongated crescent shape^11^,^12^ and constitutively interacts with Atg29 and Atg31 *in vivo*, forming a trimeric complex (Atg17^TC^ in the following)^13^,^14^. On autophagy induction, Atg1 and Atg13 assemble with Atg17^TC^ into the pentameric Atg1–kinase complex (Atg1^PC^ in the following)^13^,^15^. Interestingly, mammalian homologues of Atg1 (ULK1/2), Atg13 (ATG13) and potentially Atg17 (FIP200) have been identified^16^,^17^,^18^. Moreover, the ULK1-complex regulates mATG9 localization and is involved in early steps of autophagy, suggesting that yeast Atg1- and mammalian ULK1/2-complexes possess similar activities in autophagy^19^,^20^. Initiation of mammalian autophagy occurs, however, at specialized ER-domains, termed omegasomes^6^. Thus, although mammalian and yeast autophagy pathways share conserved elements, both are driven by distinct yet to be characterized mechanisms.

Atg1^PC^ integrates nutrient dependent signals from other kinases such as the protein kinase A (PKA) and the target of rapamycin complex (TORC) 1 (refs 21, 22). Under vegetative conditions, TORC1 phosphorylates Atg13, preventing its interaction with Atg17 (ref. 23) and thus the assembly of Atg1^PC^ from its subunits. On deactivation of TORC1 Atg13 becomes partially dephosphorylated and functional Atg1^PC^ assembles at the PAS^24^.

Significant progress has been made in the past to decipher the function of Atg1^PC^ as well as its interaction with Atg9-vesicles^12^,^23^,^25^,^26^. The molecular mechanism of Atg1^PC^-activation and how this translates into its regulated interaction with Atg9 remained, however, elusive. Here we analysed for the first time the Atg1^PC^-Atg9-interaction in a fully reconstituted *in vitro* system using purified components. Our results show that the Atg17-dimer binds two Atg9-vesicles independently and functions as vesicle-tether without contacting the membrane directly. The Atg31–Atg29 subcomplex sterically blocks the Atg9-binding site in Atg17 such that Atg17^TC^ becomes inactive. On autophagy induction, the pentameric complex Atg1^PC^ assembles and restores the full Atg9-binding and vesicle-tethering activity of Atg17.

## Results

### Reconstitution of Atg1^PC^ *in vitro*

**Figure 1:**
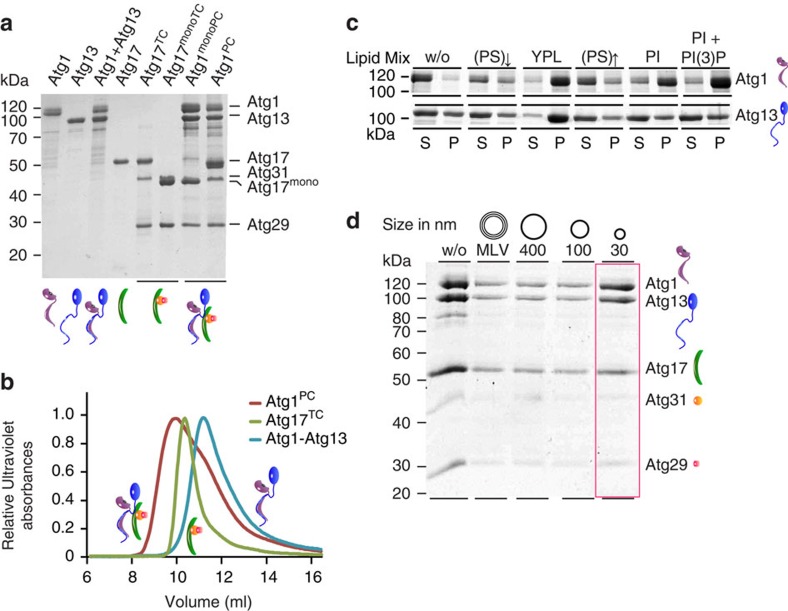
Reconstitution of Atg1^PC^ from purified components. (**a**) SDS–PAGE of recombinantly expressed and SEC-purified Atg1–kinase complex subunits. Atg17^TC^ and Atg17^monoTC^ were co-expressed; Atg1–Atg13, Atg1^PC^ and Atg1^monoPC^ were reconstituted from single components. (**b**) SEC elution profiles of Atg1^PC^ (red) compared with that of Atg17^TC^ (green) and Atg1–Atg13 subcomplexes (blue). (**c**) Liposome sedimentation assays of Atg1 and Atg13. Lipid specificity of Atg1 and Atg13 was assessed by comparing their interaction with SUV containing 10 mol% PS ([PS]↓), YPL-extracts, 40 mol% PS ([PS]↑), 30 mol% PI, or 25 mol% PI supplemented with 2.5 mol% PI(3)P. S, supernatant; P, pellet. (**d**) Floatation assay of YPL-large unilamellar vesicles (LUVs) and MLVs of different sizes with Atg1^PC^. Atg1^PC^ is efficiently recruited to membranes with high curvature (red box). Cartoons represent subunits and complexes.

To study the intricate interplay of Atg1^PC^ and Atg9-vesicles in a fully reconstituted system, we purified individual subunits and subcomplexes to incrementally assemble full-length Atg1^PC^ from its components (Fig. 1a, Supplementary Fig. 1a). Recombinant Atg1 was phosphorylated at T226 (Supplementary Fig. 1b, Supplementary Table 1), representing the active enzyme that functions in autophagy^27^,^28^. In contrast to the recombinant Atg1-MIT-domain, which dimerized in solution^12^,^25^, full-length Atg1 was monomeric (Supplementary Fig. 1c). Atg17^TC^ was produced by co-expressing Atg17, Atg31 and Atg29 or by combining Atg17 with co-expressed Atg31–Atg29. Atg17 and Atg17^TC^ form well-characterized S-shaped dimers with dimerization being mediated by the C-terminus of Atg17 (refs 11, 12). To confirm that our recombinant Atg17 and its related complex form similar dimers, we determined their Stokes radii (*R*_*H*_) using dynamic light scattering (DLS). Atg17 and Atg17^TC^ possessed very similar *R*_*H*_ of 10.4±0.1 nm and 10.3±0.1 nm (±refers to s.d., *n*=3), respectively. Thus, Atg17 and Atg17^TC^ form extended dimers comparable to those observed previously^11^. Correspondingly, the C-terminally truncated variants Atg17^mono^ (Atg17^1–354^) and Atg17^monoTC^, which lack the dimerization domain, are monomeric (*R*_H_=5.3±0.1 nm and 5.6±0.2 nm,±refers to s.d., *n*=5).

Atg1 and Atg13 formed a stable subcomplex and incubating both with Atg17^TC^ resulted in the formation of Atg1^PC^ (Fig. 1a,b, Supplementary Fig. 1d). Multi-angle light scattering (MALS) revealed that Atg1^PC^ is a dimer of pentamers (Supplementary Fig. 1e). Although the Atg1–Atg13 subcomplex contributes two-thirds of the total mass of Atg1^PC^, the *R*_H_ (∼13 nm) of Atg1^PC^ is only slightly larger than that of Atg17^TC^. Atg1, Atg13 and Atg29 have been reported to contain intrinsically disordered regions that are involved in intermolecular interactions that stabilize Atg1^PC^ (refs 29, 30). Moreover, previous electron microscopy studies showed that the overall S-shaped structure of Atg17^TC^ was not perturbed upon binding of the C-terminal domains of Atg1 and Atg13 (ref. 30). These observations are in agreement with our data demonstrating that Atg1^PC^ has similar overall hydrodynamic properties as Atg17^TC^. Moreover, our results show that N-terminal domains of Atg1 and Atg13 do not significantly increase the *R*_H_ of the complex, suggesting that Atg1 and Atg13 form a compact subcomplex that partially occupies the Atg17-crescent. The monomeric variant Atg17^monoTC^ assembled with Atg1–Atg13 into monomeric Atg1^PC^ (Atg1^monoPC^) as demonstrated by a shift in the retention volume (*V*_R_) from 10.2 ml (Atg1^PC^) to 12.6 ml (Atg1^monoPC^) in size exclusion chromatography (Supplementary Fig. 1f). Thus, complex formation and Atg17-mediated dimerization are independent of each other.

### Atg1 and Atg13 are membrane-binding proteins

The C-terminal tandem MIT-domain of yeast Atg1 has been found to bind highly curved membranes *in vitro*^12^. To what extend full-length Atg1 targets fully assembled Atg1^PC^ to membranes remained, however, elusive.

To address this question, we analysed membrane binding of individual Atg1^PC^-components. While Atg17 and Atg17^TC^ did not interact with membranes of any curvature, both Atg1 and Atg13 were efficiently recruited to highly curved membranes composed of yeast polar lipid (YPL) extracts (Supplementary Fig. 1g).

To test whether specific lipids are recognized by Atg1 and Atg13, we compared binding to small unilamellar vesicles (SUVs) made of YPL-extracts with that to SUVs containing synthetic lipid mixtures. Acidic SUVs containing 10 mol% or 40 mol% phosphatidylserine (PS) did not facilitate Atg1 or Atg13 recruitment. Interestingly, however, Atg1 strongly bound phosphatidylinositol (PI)-containing SUVs. Replacing 5 mol% PI by PI(3)-phosphate (PI(3)P) enhanced Atg1 recruitment further (Fig. 1c). By contrast, Atg13 was only recruited to SUVs prepared from YPL-extracts, suggesting that other lipids than those present in tested synthetic mixtures are required for Atg13 binding (Fig. 1c). We next investigated whether full-length Atg1^PC^ is targeted to membranes by Atg1 and Atg13. Using floatation assays we found similar membrane-binding characteristics of Atg1^PC^ (Fig. 1d) compared with those of Atg1 (Supplementary Fig. 1g). The specificity of Atg1 for PI-containing membranes of high curvature is consistent with the localization of Atg1^PC^ *in vivo*. Atg1^PC^ is one of the earliest complexes to assemble at the PAS followed by Atg13-mediated recruitment of the autophagy specific PI(3)-kinase complex^31^. The conversion of PI to PI(3)P within autophagic membranes occurs, thus, downstream of Atg1 (ref. 32).

### Atg17 binds the conserved Atg9-core to promote autophagy

**Figure 2:**
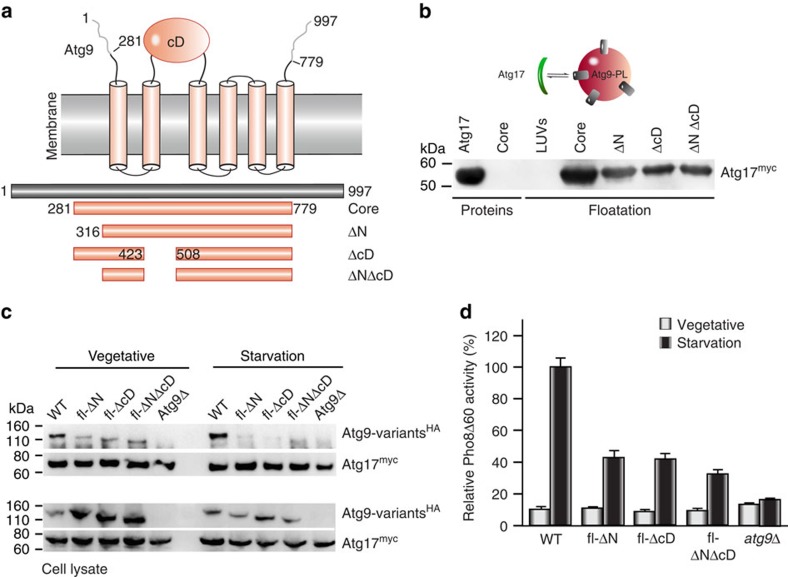
Cytoplasmic domains of Atg9 interact with Atg17 to promote autophagy. (**a**) The scheme shows the topology of Atg9 in membranes (transmembrane domains, pink cylinders; membrane bilayer in grey; cD, cytoplasmic domain). The grey bar represents Atg9^WT^, the pink bars illustrate Atg9-variants with corresponding domain borders indicated by numbers. (**b**) α-myc immunoblot of co-floatation assays of Atg9-PL, containing Atg9^core^ or its variants, with Atg17. Large unilamellar vesicles (LUVs) lacking Atg9 served as a control for unspecific membrane binding. Proteins, recombinant Atg17 and Atg9–PLs used for experiments. (**c**) Immunoprecipitation (IP) of Atg17^myc^ from *ATG9*^WT^, *ATG9*^ΔN^, *ATG9*^ΔcD^, *ATG9*^ΔNΔcD^ and *ATG9*Δ lysates under vegetative conditions and after 2 h of starvation. *Atg9*-variants contained the very N- (1–280) and C-terminal (779–997) unstructured regions, which were not present in Atg9^core^ (indicated by the prefix fl), because these regions are essential for proper trafficking of Atg9 *in vivo*^10^,^49^,^50^. Atg17 and co-immunoprecipitated Atg9-variants were detected by α-myc and α-HA immunoblots as indicated. Atg17 strongly interacts with wild-type Atg9, but not with Atg9 lacking N-, cD- or both domains. Bottom panel: total protein amounts in cell lysates. (**d**) Pho8Δ60 assay of vegetative (white bars) and starved (black bars) *ATG9*^WT^, *ATG9*^ΔN^, *ATG9*^ΔcD^, *ATG9*^ΔNΔcD^ and *ATG9*Δ. The Pho8Δ60 activity of starved *ATG9*^WT^ was set to 100% and used for normalization. Mean values±s.d. of *N*=3 independent experiments are shown.

During autophagy, Atg1^PC^ assembles at the PAS and recruits Atg9-vesicles in order to initiate the formation of autophagosomes^10^,^33^. Thus lipid-binding as observed for Atg1 and Atg13 needs to be complemented by a specific recognition of Atg9. Atg17 is the first Atg1^PC^-subunit to arrive at the PAS on induction of autophagy^7^, which is followed by Atg17-dependent recruitment of Atg9-vesicles^34^. We therefore aimed to compare binding of Atg17, Atg17^TC^ and Atg1^PC^ to Atg9-vesicles in a fully reconstituted system using purified components. Atg9 possesses in addition to weakly conserved and mostly disordered N- and C-terminal regions a highly conserved core region (Atg9^core^ in the following), which corresponds to the ubiquitously expressed metazoan Atg9A-orthologue. We therefore focused on Atg9^core^ (Fig. 2a) and studied its interaction with Atg1^PC^.

We first confirmed that Atg9^core^ retained its capacity to bind Atg17 *in vivo* by co-immunoprecipitating HA-tagged Atg9^core^ with Atg17^GFP^ (Supplementary Fig. 2a). Next, we reconstituted recombinant Atg9^core^ in proteoliposomes (Atg9–PLs). Circular dichroism (CD) spectroscopy of detergent-solubilized and purified Atg9^core^ revealed the protein to be correctly folded (secondary structure content of 77.5%), possessing an α-helix content of 46.2% (Supplementary Fig. 2b), which is comparable to that of similar membrane proteins^35^. Assessing the orientation of reconstituted Atg9^core^ in PLs by protease protection revealed that ∼50% exposed their cytoplasmic domain to the exterior, adapting the native membrane topology (Supplementary Fig. 2c).

We next tested whether Atg17 directly interacts with Atg9–PLs and found that Atg17 was indeed recruited to Atg9–PLs, but not to liposomes lacking Atg9 in co-floatation assays (Fig. 2b, Supplementary Fig. 2d). To determine which region in Atg9 contributes to Atg17-recognition, we further truncated Atg9^core^ to produce deletion variants in which the entire N-terminal domain (Atg9^ΔN^), the cytoplasmic domain between transmembrane helix two and three (Atg9^ΔcD^), or both domains (Atg9^ΔNΔcD^) were missing (Fig. 2a). CD-spectroscopy confirmed that all recombinant Atg9-variants were correctly folded (Supplementary Fig. 2b). In contrast to Atg9^core^, which did not prefer a specific orientation, Atg9-variants were incorporated such that their cytoplasmic domains were facing the exterior. The orientation of Atg9-variants in PLs is thus similar to the native topology of Atg9 *in vivo*. (Supplementary Fig. 2c). As a consequence, the amount of accessible binding sites is larger than that of equal amounts of liposomes containing Atg9^core^. Even though more binding sites are available in PLs containing Atg9-variants, a strong reduction in co-floating Atg17 was observed (Supplementary Fig. 2d). To eliminate the interference of Atg9 on the intensity of Atg17-signals, we quantified co-floating myc-tagged Atg17 (Atg17^myc^) by immunoblotting (Fig. 2b, Supplementary Fig. 2e). All Atg9-variants were strongly impaired in recruiting Atg17 by >60%, indicating that both N-terminal and cytoplasmic domains of Atg9 are crucial for Atg17-binding.

To investigate binding of Atg17 to Atg9 and its variants *in vivo*, we complemented yeast *atg9Δ* cells with *atg9*^WT^, *atg9*^ΔN^ (Δ281–315), *atg9*^ΔcD^ (Δ424–507), and *atg9*^ΔNΔcD^ in trans. We first analysed whether Atg9-variants are forming peripheral Atg9-pools using subcellular fractionation as previously reported^33^ and found distribution-profiles of Atg9-variants under autophagy-induced conditions to be comparable to that of Atg9^WT^ under vegetative conditions (Supplementary Fig. 2f). We next studied binding of Atg17 to Atg9. As reported previously^34^, Atg17 co-immunoprecipitated Atg9 even under vegetative conditions and inducing autophagy by starvation strongly enhanced their interaction (Fig. 2c). Consistent with our *in vitro* data, we observed a strongly decreased interaction of Atg17 with Atg9^ΔN^, Atg9^ΔcD^ and Atg9^ΔNΔcD^ under vegetative conditions, which was entirely abolished on starvation (Fig. 2c). We inferred from these results that due to the missing interaction of Atg17 with Atg9-variants, Atg9-vesicles are not recruited from peripheral pools to the PAS, which is consistent with previous observations^7^,^34^. Consequently, autophagic activity which we quantified using Pho8Δ60-assays was strongly decreased in *atg9*^ΔN^, *atg9*^ΔcD^, and *atg9*^ΔNΔcD^ cells (Fig. 2d). Our data thus demonstrate that both N- and cD-domains are required for Atg17-binding to facilitate efficient autophagy.

### Competition between Atg9 and Atg31 regulates Atg17 activity

**Figure 3:**
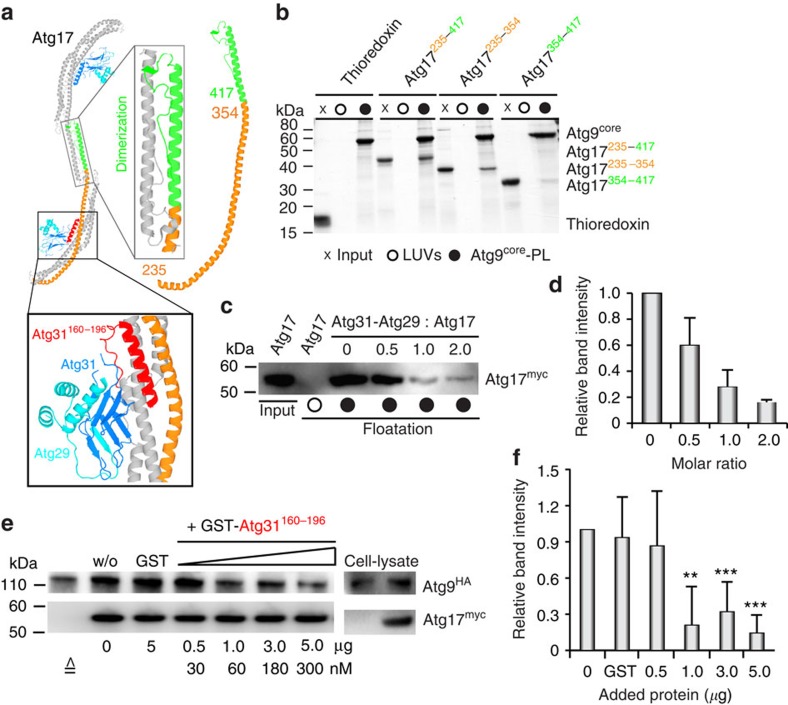
Atg9 binds the central crescent of Atg17. (**a**) Cartoon representation of the crystal structure of Atg17^TC^ (pdb 4HPQ). Helix α4 (orange and green) spans the entire Atg17-crescent and forms part of the dimerization domain (green). Domain borders that have been used to design Atg17-variants are indicated by numbers. The minimal Atg17-binding region of Atg31 is highlighted in red with numbers indicating corresponding domain borders. Images were prepared in PYMOL (The PyMOL Molecular Graphics System, Version 1.2r3pre, Schrödinger, LLC). (**b**) SDS–PAGE gel of co-floatation experiments of Atg9–PLs with recombinant thioredoxin-tagged Atg17-fragments. Thioredoxin-tag lacking Atg17 (w/o Atg17) and co-floatation with large unilamellar vesicles (LUVs) lacking Atg9^core^ served as controls. Input corresponds to 10% of total protein used for co-floatation. (**c**) α-myc immunoblot of co-floatation experiments of Atg17 with Atg9–PLs and increasing stoichiometric ratios of Atg31–Atg29. After incubating Atg9–PLs with Atg17, purified Atg31–Atg29 was added. Atg31–Atg29 inhibits binding of Atg17 to Atg9 *in vitro*. (**d**) Quantification of Atg17 retrieved from the floating fraction in co-floatation experiments with Atg9–PLs as shown in **c**. The intensity of the Atg17-band in the absence of Atg31–Atg29 was set to one and used for normalization. Mean values±s.d. of *N*=3 independent experiments are shown. (**e**) Immunoprecipitation (IP) of Atg17^myc^ from cell lysates after 2 h of starvation. Increasing amount of GST-Atg31^160–196^ as indicated or 5 μg GST were added to cell lysates prior IP. Atg17 and co-immunoprecipitated Atg9 were detected by α-myc and α-HA immunoblots. (**f**) Quantification of Atg9^HA^ as shown in **e**. The intensity of the Atg9^HA^-band (w/o GST-Atg31^160–196^) was set to one and used for normalization. Mean values±s.d. of *N*=3 independent experiments are shown. *P* values were calculated using two-tailed Student's *T*-test (*P<0.05; **P<0.01; ***P<0.005).

We identified a stable C-terminal degradation product of Atg17, which apparently retained the capacity to interact with Atg9. This fragment contained the entire helix α4 of Atg17 and its C-terminal dimerization-domain (Fig. 3a). The corresponding recombinantly expressed Atg17-fragment (Atg17^235–417^) was dimeric as expected. Moreover, co-floatation experiments demonstrated that Atg17^235–417^ indeed strongly interacted with Atg9–PLs (Fig. 3b). To further narrow down the potential Atg9-binding site in Atg17, we expressed Atg17^235–354^ (lacking the dimerization domain, thus being monomeric) and Atg17^354–417^ (dimerization domain) separately. Although both constructs bind Atg9–PLs to a certain extent, binding of Atg17^235–354^ was much stronger than that of Atg17^354–417^, suggesting that the N-terminal part of α4 harbours the primary binding site for Atg9 (Fig. 3b). Interestingly, Atg31 occupies a similar region in Atg17 (ref. 36; Fig. 3a). To investigate whether Atg31 and Atg9 compete with each other, we performed co-floatation experiments of Atg17 and Atg9–PLs adding increasing stoichiometric amount of purified Atg31–Atg29 subcomplex (Fig. 3c). Remarkably, Atg31–Atg29 strongly inhibited Atg17 for Atg9-binding. In the presence of equimolar amounts of Atg17 and Atg31–Atg29, only ∼30% of Atg17 was recovered from the Atg9-PL fraction (Fig. 3d).

The interaction interface between Atg17 and its regulatory Atg31–Atg29 subcomplex is composed of two C-terminal α-helices of Atg31 (residues 160–196), which form a helical bundle with Atg17 (Fig. 3a). We therefore predicted that Atg31^160–196^ efficiently inhibits binding of Atg17 to Atg9 *in vivo* given that a similar molecular mechanism as observed in our reconstituted system regulates Atg17 activity in yeast. To test this hypothesis, we co-immunoprecipitated Atg9^HA^ with Atg17^myc^ from cell lysates which were incubated with increasing amounts of recombinant, purified GST-Atg31^160–196^ (Fig. 3e). We observed a strong and concentration-dependent decrease in co-immunoprecipitated Atg9 (Fig. 3e), resulting in ∼20% residual binding on incubation with one to five μg GST-Atg31^160–196^, which amounts to concentrations between 60 and 300 nM GST-Atg31^160–196^ in cell lysates (Fig. 3f). The estimated concentration of Atg17 in cell lysates (based on a Atg17-copy number of ∼500 per cell^26^ and 50 OD yeast cells in 500 μl lysate) is ∼15 nM. Thus Atg31^160–196^ is a strong competitive inhibitor of the Atg17–Atg9 interaction. The residual ∼20% binding activity might be attributed to an Atg17-independent interaction of Atg1^PC^ with Atg9, which has recently been found to be mediated by Atg13 (ref. 14). We next confirmed that the inhibitory effect of GST-Atg31^160–196^ is caused by direct and competitive interaction with Atg17 by co-immunoprecipitating Atg17 with GST-Atg31^160–196^ (Supplementary Fig. 3). In summary, our *in vitro* and *in vivo* data show that the Atg31–Atg29 subcomplex regulates binding of Atg17 to Atg9. Moreover, the direct physical interaction between Atg17 and Atg9 appears to dominate other interactions of Atg1^PC^-subunits with Atg9 *in vivo*, since Atg31^160–196^ strongly inhibits binding of Atg17 to Atg9. Thus, the interaction of Atg1^PC^ with Atg9-vesicles appears to be controlled by a direct and tightly regulated binding of the scaffolding subunit Atg17 to Atg9 *in vitro* and *in vivo*.

### The Atg1–Atg13 subcomplex activates Atg17^TC^

**Figure 4:**
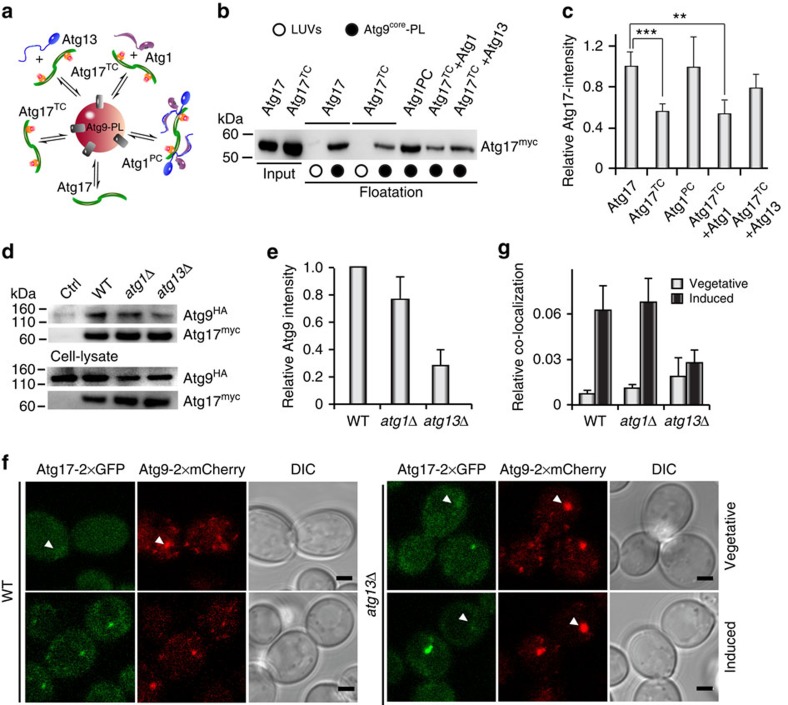
The activity of Atg17 is regulated by Atg31–Atg29 and Atg1–Atg13 subcomplexes *in vitro* and *in vivo.* (**a**) The schematic drawing illustrates the experimental set-up of floatation assays. (**b**) α-myc immunoblot from co-floatation experiments of Atg9–PLs with recombinant myc-tagged Atg17 with and without other Atg1^PC^-subunits as indicated. Similar experiments with large unilamellar vesicles (LUVs) lacking Atg9^core^ served as controls for unspecific membrane binding. Input corresponds to 10% of total protein used for co-floatation. (**c**) Semiquantitative analysis of immunoblots as shown in **b** from co-floatation experiments of Atg17^myc^ with Atg9–PLs in the presence of other subunits of Atg1^PC^ as indicated. All Atg17^myc^-intensities were corrected for Atg9-intensities. The band intensity of Atg17^myc^ (w/o other Atg1^PC^-subunits) was set to one and used for normalization. Mean values±s.d. of *N*=3 independent experiments are shown. *P* values were calculated using two-tailed Student's *T*-test (*P<0.05; **P<0.01; ***P<0.005). (**d**) Immunoprecipitation (IP) of Atg17^myc^ from lysates of wildtype (WT), *atg1*Δ and *atg13*Δcells after two hours of starvation. Atg17 and co-immunoprecipitated Atg9 were detected by α-myc and α-HA immunoblots as indicated. (**e**) Quantification of Atg9^HA^ as shown in **d**. The intensity of the Atg9^HA^-band in wildtype (WT), *atg1*Δ, and *atg13*Δ cells were corrected for unspecific binding of Atg9^HA^ in the control (Ctrl, cells w/o myc-tag). The WT Atg17-band intensity was set to one and used for normalization. Mean values±s.d. of *N*=3 independent experiments are shown. (**f**) Co-localization of Atg9-tandem-mCherry with Atg17-tandem-GFP in wildtype (WT) and *atg13*Δ cells under vegetative and autophagy-induced conditions. Arrowheads indicate positions of non-colocalizing Atg9-puncta. Scale bar, 2 μm. (**g**) Quantification of relative co-localization of Atg17-puncta with Atg9-puncta (number of co-localizing puncta per cell, normalized by number of Atg17 puncta per cell) in WT, *atg1*Δ, and *atg13*Δ cells under vegetative (white bars) and autophagy-induced (black bars) conditions. Mean values±s.d. of *N*=3 independent experiments are shown (>200 cells per strain were examined).

Under vegetative conditions, most of Atg17^TC^ resides in the cytoplasm in its inactive state. On starvation, however, Atg1–Atg13 associates with Atg17^TC^ at the PAS to initiate autophagy. In context of the observed competition between Atg9 and Atg31–Atg29, we predicted that Atg1–Atg13 activates Atg17^TC^ to restore Atg9-binding. We thus compared binding of Atg17, Atg17^TC^ and Atg1^PC^ with Atg9–PLs (Fig. 4a).

Atg9–PLs used in this study were prepared from synthetic lipid mixtures lacking PI. Consequently, Atg1 and Atg13 were not recruited to Atg9–PLs in the absence of Atg17 or Atg17^TC^ (Supplementary Fig. 4a). Consistent with our competition assay, co-purified Atg17^TC^ was strongly impaired in Atg9 binding. However, combining Atg1–Atg13 with Atg17^TC^ fully restored the binding capacity of Atg17 (Fig. 4b,c, Supplementary Fig. 4b). In agreement with our and other data that only Atg13 directly contacts Atg17, Atg1 alone did not and Atg13 alone partially activated Atg17^TC^ for Atg9 binding (Fig. 4b,c).

To confirm that this mechanism of activation regulates Atg17 activity *in vivo* and thus autophagy initiation, we co-immunoprecipitated Atg9^HA^ with Atg17^myc^ from cell lysates of starved wildtype, *atg1*Δ, and *atg13*Δ cells (Fig. 4d). Consistent with our observations *in vitro*, deletion of *atg1* had a minor impact on the interaction of Atg17 and Atg9. In the absence of Atg13, however, a reduction in co-immunoprecipitated Atg9 by ∼70% was observed (Fig. 4e). In conclusion our experiments suggest that the constitutive Atg17-Atg31–Atg29 complex^7^,^15^ is inactivated by occlusion of the Atg9-binding site. On autophagy induction, Atg17^TC^ recruits Atg1–Atg13 to the PAS^7^,^15^ and their direct interaction releases the inhibition of Atg17 to restore its full Atg9-binding capacity.

The recruitment of Atg9-vesicles by Atg17 to the PAS is one of the earliest steps in autophagy. We thus investigate whether regulation impacts on the co-localization of the two proteins using wildtype, *atg13*Δ and *atg1*Δ cells. In the wild-type background, multiple Atg17-negative Atg9-puncta were detected under vegetative conditions and inducing autophagy increased the relative co-localization of both proteins drastically (Fig. 4f,g). A similar co-localization was observed in *atg1*Δ cells, arguing that Atg13 itself sufficiently activates Atg17^TC^ (Fig. 4g, Supplementary Fig. 4c) By contrast, autophagy induction did not promote co-localization of Atg17 and Atg9 in *atg13*Δ cells, demonstrating that inhibited Atg17^TC^ is strongly impaired in recruiting Atg9-vesicles to the PAS (Fig. 4f,g).

### The dimerization domain of Atg17 is involved in regulation

**Figure 5:**
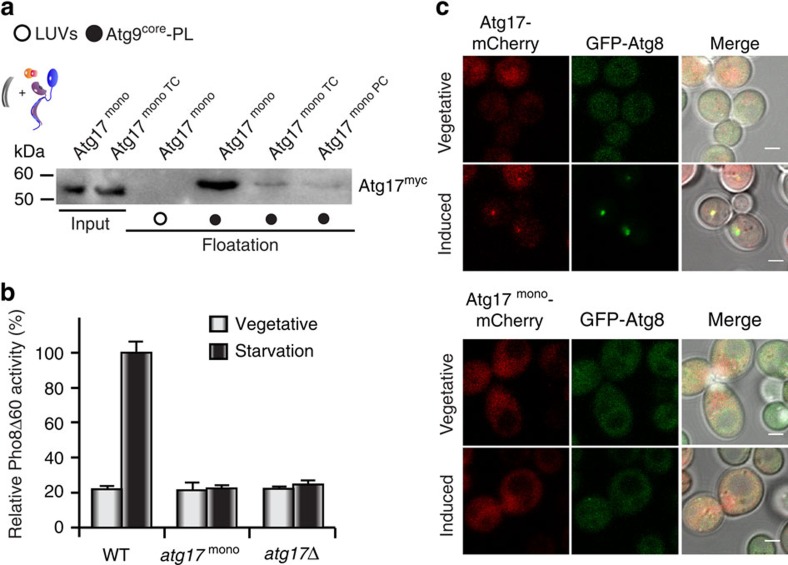
The dimerization domain of Atg17 regulates Atg17-function. (**a**) α-myc immunoblots from co-floatation experiments of Atg9–PLs with recombinant myc-tagged, monomeric Atg17 (Atg17^mono^), Atg17^monoTC^ and Atg1^monoPC^. Atg17^mono^ is strongly inhibited by Atg31–Atg29, but Atg17^monoTC^ is not activated by Atg1–Atg13. Similar experiments with large unilamellar vesicles (LUVs) lacking Atg9^core^ served as controls. Input corresponds to 10% of total protein used for co-floatation. (**b**) Pho8Δ60 assay of wildtype (WT), *atg17*^mono^, and *atg17*Δ cell lysates to assess autophagic activity under vegetative (grey bars) and starvation (black bars) conditions. Mean values±s.d. of *N*=3 independent experiments are shown. (**c**) Co-localization of GFP-Atg8 with mCherry-tagged Atg17 or Atg17^mono^ under vegetative or autophagy-induced conditions. Atg17^mono^ is not forming puncta under autophagy-induced conditions. Consequently, autophagosomes are not being formed as apparent from the lack of Atg8-puncta. Scale bar, 2 μm.

Our observation that the dimerization domain of Atg17 is dispensable for Atg9-binding (Fig. 3b) prompted us to investigate, whether monomeric Atg17 is regulated in a similar manner as observed for full-length Atg17. We thus compared binding of Atg17^mono^, Atg17^monoTC^ and Atg17^monoPC^ with Atg9-PL using floatation assays. Consistent with our data that helix α4 of Atg17 harbours the primary Atg9-binding site, Atg17^mono^ was efficiently recruited to Atg9–PLs (Fig. 5a). Remarkably, Atg17^mono^ was much stronger inhibited by Atg31–Atg29 as it has been observed for Atg17. Moreover, Atg1–Atg13 was not able to release this strong inhibition (Fig. 5a), although both subcomplexes assemble into stable Atg1^monoPC^ (Supplementary Fig. 1f). Thus, the dimerization domain of Atg17 is apparently involved in regulating Atg17-function. The strong inhibition of Atg1^monoPC^ also demonstrates that membrane binding of Atg1 and Atg13 *per se* does not contribute to enhanced binding of full-length Atg1^PC^ to Atg9–PLs.

We next tested whether the residual Atg9-binding activity of Atg17^monoTC^ and Atg1^monoPC^ suffices to induce autophagy. Consistent with previous findings^12^, autophagy is strongly impaired in cells expressing Atg17^mono^ (Fig. 5b). This earlier study also demonstrated that the autophagy-defect was caused by an impaired recruitment of Atg8 to the PAS^12^. Since Atg17 functions upstream of Atg8, we investigated whether Atg17^mono^ localizes to the PAS by analysing mCherry-Atg17- and GFP-Atg8-puncta formation in cells expressing Atg17 or Atg17^mono^ (Fig. 5c). We performed these experiments in *atg11*Δ cells to prevent Cvt-mediated GFP-Atg8-puncta formation. As expected, both Atg17 and Atg8 were recruited to the PAS in cells expressing full-length Atg17 under autophagy-induced conditions. Interestingly, however, neither Atg8- nor Atg17-puncta were observed in Atg17^mono^ expressing cells. (Fig. 5c). Thus, expression of Atg17^mono^ inhibits early steps in autophagy, arguing that dimerization of Atg17 itself is important for its proper localization and function *in vivo*.

### Atg1^PC^ is a membrane-tethering complex

Our data revealed the mechanism that regulates Atg17 activity, implying that dimerization of Atg17 is important for its targeting to and activation at the PAS. Moreover, the S-shaped structure^11^,^12^ of Atg17^TC^ suggests that one Atg17-dimer binds two Atg9-vesicles independently. We thus tested whether Atg17 has membrane-tethering activity by measuring the *R*_*H*_ of Atg9–PLs in the presence and absence of Atg17 using DLS.

**Figure 6:**
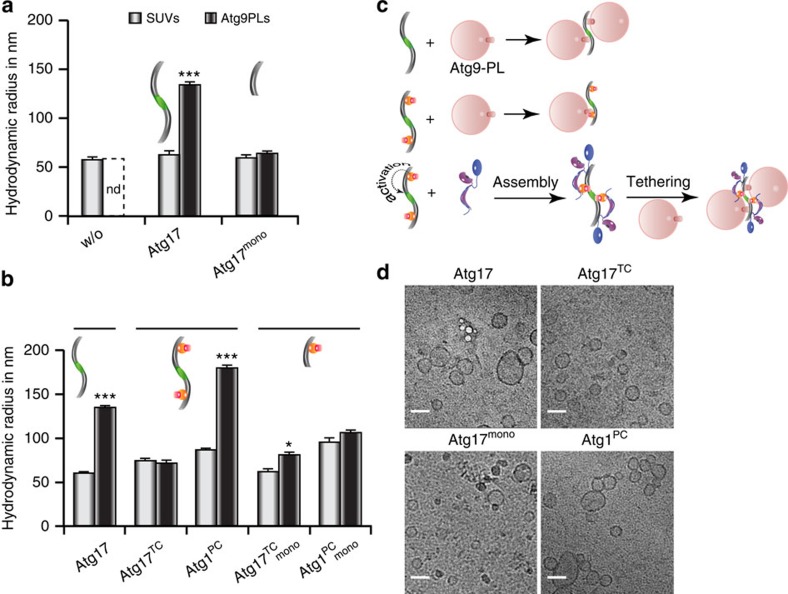
Atg1^PC^ tethers Atg9–PLs. (**a**) The hydrodynamic radius of SUVs (white bar) and Atg9–PLs (black bar) obtained from DLS-experiments in the absence or presence of Atg17 and corresponding variants as illustrated by cartoons are shown. ND, not determinable (polydisperse). (**b**) Similar experiments shown in **a** were performed for Atg17 and Atg17-variants and that of corresponding Atg17^TC^ and Atg1^PC^ (sub)complexes. (**a**,**b**). Mean values±s.d. of *N*=5 experiments are shown for both **a** and **b**. *P* values were calculated using two-tailed Student's *T*-test (*P<0.05; **P<0.01; ***P<0.001). (**c**) The schematic drawing illustrates the experimental setup and summarizes the results obtained from DLS-experiments in **a** and **b**. (**d**) Cyro-electron micrographs of Atg9–PLs from samples used for DLS-experiments containing Atg17, Atg17^TC^, Atg17^mono^, and Atg1^PC^. Atg17 tethers two and Atg1^PC^ tethers multiple vesicles together. Scale bar, 50 nm.

Atg9–PLs are polydisperse in solution, indicating that they are clustering. This is consistent with previous data showing that Atg9 possesses a highly conserved self-interacting motif that is required for Atg9-trafficking and function *in vivo*^37^. To circumvent Atg9-mediated self-interaction, we prepared Atg9–PLs in the presence of either Atg17 (Atg9–Atg17–PLs) or Atg17^mono^. In contrast to SUVs lacking Atg9 (*R*_H_=60±2 nm, Fig. 6a), Atg9–Atg17–PLs possessed an *R*_H_ of 135±2 nm (±refers to s.d., *n*=5), indicating two Atg9–PLs to be tethered together by dimeric Atg17. To exclude that other effects than specific tethering cause Atg9–PLs to appear larger by DLS, we analysed Atg17^mono^ for its tethering activity. The size of Atg9–Atg17^mono^–PLs (*R*_H_=65±1 nm, (refers to s.d., *n*=5) was similar to that of corresponding SUVs lacking Atg9^core^ (Fig. 6a). Thus, dimerization of Atg17 is required to tether Atg9–PLs.

Atg17 constitutively interacts with Atg31–Atg29 *in vivo* and we demonstrated that Atg17 is inhibited by Atg31–Atg29 for Atg9-binding *in vitro* and *in vivo* (Fig. 3c,d). We therefore tested whether this inhibition impacts on tethering. Atg9–PLs, prepared in the presence of Atg17^TC^ and Atg17^monoTC^ were monodisperse with a *R*_H_ of 72±2 nm and 82±2 nm (±refers to s.d., *n*=5), respectively (Fig. 6b,c). Thus Atg31–Atg29 does not only interfere with Atg9-binding, it also regulates tethering of Atg9–PLs by Atg17. Consequently, Atg1–Atg13-mediated activation of Atg17^TC^ should promote membrane-tethering by Atg1^PC^. In fact, Atg9–Atg1^PC^-PLs were found to be twice the size (*R*_H_=180±4 nm) of corresponding SUVs lacking Atg9^core^ (*R*_*H*_=87±2 nm,±refers to s.d., *n*=5) (Fig. 6b,c). Consistent with our data on Atg17^TC^-regulation (Fig. 4c), Atg1 alone did not promote tethering of Atg9–PLs by Atg17^TC^. In the presence of Atg13, however, Atg9–Atg17^TC^–PLs were significantly larger (*R*_H_=105±5 nm) and polydisperse (22±3% compared to 12–16% in other samples,±refers to s.d., *n*=5), indicating that a mixture of tethered and dispersed vesicles is present in these samples (Supplementary Fig. 5a). Thus, Atg13 partially activates Atg17^TC^, which allows the resulting complex to tether Atg9–PLs, but with lower efficiency compared with Atg17 or Atg1^PC^. Furthermore, Atg1^monoPC^ was deficient in tethering Atg9–PLs (Fig. 6b), demonstrating that dimerization and activation of Atg17 are required to tether Atg9-vesicles.

Since DLS is an indirect measurement for vesicle tethering, we set out to visualize this process by cryoelectron microscopy. We found that Atg9–Atg17–PLs are 20–50 nm in diameter (Fig. 6d, Supplementary Fig. 5b,c), closely resembling the size of Atg9-vesicles that are recruited to the PAS to nucleate the phagophore *in vivo*^10^. Most importantly, however, the majority of such vesicles was found to be tethered, resulting in two vesicles contacting each other (Fig. 6d, Supplementary Fig. 5b,d). Consistent with our DLS measurements, Atg9–Atg17^mono^–PLs and Atg9–Atg17^TC^–PLs were homogeneously dispersed with diameters similar to that of Atg9–Atg17–PLs (Supplementary Fig. 5b,c). In contrast, multiple Atg9–PLs were contacting each other in the presence of Atg1^PC^ (Fig. 6d, Supplementary Fig. 5b, d). Moreover, Atg1^PC^-mediated tethering progressed from two vesicles to multiple vesicles, resulting in a steady increase in *R*_H_ and polydispersity in DLS measurements over time. Interestingly, an average of three Atg9-vesicles have been shown to coalesce at the PAS to nucleate the phagophore^10^, which is consistent with our finding that Atg1^PC^ tethers multiple vesicles.

### Phosphorylated Atg13 is deficient in activating Atg17^TC^

Under vegetative conditions, Atg13 is phosphorylated by TORC1 (ref. 24), preventing its interaction with Atg17 and thus the assembly of Atg1^PC^ at the PAS^13^,^23^. In order to recapitulate phosphoregulation *in vitro*, we made use of the kinase activity of recombinant Atg1. By adding ATP to fully assembled Atg1^PC^, the electrophoretic mobility of Atg13, Atg29 and Atg1 shifted on SDS–PAGE gels, indicating these proteins to be phosphorylated by Atg1 (Supplementary Fig. 6a). We thus determined the phosphorylation sites by mass spectrometry. Surprisingly, many previously described regulatory sites, which are phosphorylated in response to nutrient conditions *in vivo*, are phosphorylated by Atg1–kinase *in vitro* (Supplementary Table 1). The observed phosphorylation patterns *in vitro* did, however, not recapitulate starvation-induced regulation *in vivo* since Atg29 is phosphorylated, but Atg13 is dephosphorylated on autophagy induction^24^,^29^.

**Figure 7:**
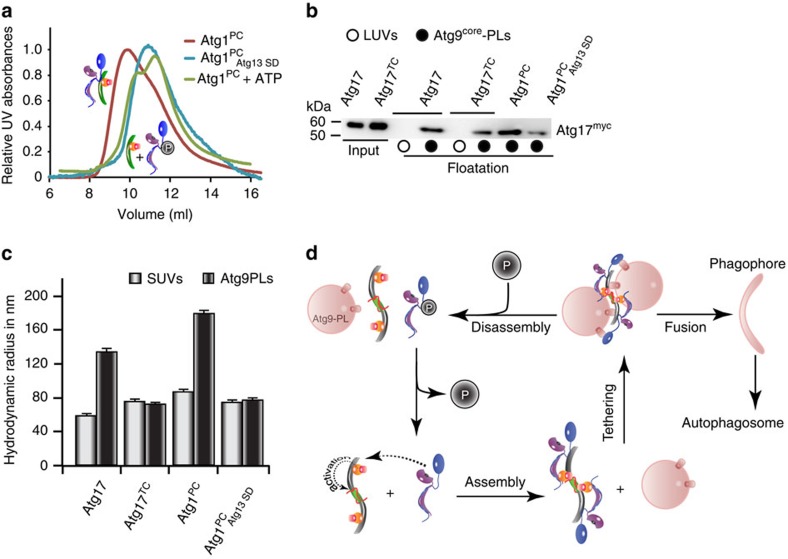
Phosphorylation of Atg13 disassembles Atg1^PC^ and prevents tethering. (**a**) SEC elution profiles of Atg17^TC^ in the presence of Atg1–Atg13 (red), Atg1–Atg13^SD^ (blue), and Atg1–Atg13+ATP (green). Atg13^SD^ and addition of ATP prevents assembly of Atg1^PC^. (**b**) α-myc immunoblot of co-floatation experiments of Atg9–PLs with recombinant myc-tagged Atg17 and Atg17^TC^ without and with Atg1–Atg13 (Atg1^PC^) or Atg1–Atg13^SD^ (ref. 23). Similar experiments with large unilamellar vesicles (LUVs) lacking Atg9^core^ served as controls. Input corresponds to 10% of total protein used for co-floatation. (**c**) The hydrodynamic radius of SUVs (white bar) and Atg9–PLs (black bar) with similar samples as shown in (**b**) obtained from DLS-experiments. Mean values±s.d. of *N*=5 experiments. (**d**) Model for Atg1^PC^ function, tethering of Atg9-vesicles, and its regulated assembly from and disassembly into subcomplexes. P indicates phosphorylation of Atg13 under vegetative conditions.

We next tested whether Atg1^PC^ is disassembled on phosphorylation. In fact, SEC and SDS–PAGE of SEC-fractions demonstrated that Atg1^PC^ dissociated on phosphorylation into Atg17^TC^ and Atg1–Atg13 (Fig. 7a, Supplementary Fig. 6b,c). Interestingly, although Atg13 was phosphorylated at several positions including serine (S)496 within the C-terminal MIM, which contacts Atg1, the Atg1–Atg13(P) subcomplex remained tightly associated. Consistent with previous findings that phosphorylation of S428 and S429 in Atg13 prevents its interaction with Atg17 (ref. 23), both residues were phosphorylated on ATP-addition to Atg1^PC^.

As indicated above, phosphorylation of Atg1, Atg13 and Atg29 is caused by promiscuous Atg1–kinase activity *in vitro*. To investigate the specific impact of Atg13-phosphorylation on Atg17-regulation, we therefore made use of the recently described phosphomimic mutant Atg13^S428D,S429D^ (Atg13^SD^)^23^. Consistent with this study, SEC-profile and SDS–PAGE of SEC-fractions demonstrate that the Atg1–Atg13^SD^ subcomplex does not interact with Atg17^TC^ (Fig. 7a, Supplementary Fig. 6d). Moreover, Atg1–Atg13^SD^ did not activate Atg17^TC^ for Atg9-binding (Fig. 7b). Consequently and consistent with all data in our study, Atg1–Atg13^SD^ did not promote tethering of Atg9–PLs by Atg17^TC^ (Fig. 7c).

In summary, our *in vitro* reconstitution study revealed important aspects of Atg1^PC^-function, including its assembly, regulation, disassembly, as well as its interaction with and tethering of Atg9-vesicles.

## Discussion

The Atg1–kinase complex assembles at the PAS upon dephosphorylation of Atg13 (refs 13, 24), where it extensively regulates autophagosome biogenesis^38^. However, the molecular function of Atg1^PC^ during phagophore biogenesis, its regulation as well as its intricate interplay with Atg9 remained controversial.

In this study, we revealed the molecular mechanism of Atg1^PC^-activation and how this impacts on its interaction with Atg9-vesicles. The key elements of this study comprise: (1) reconstitution of full-length Atg1^PC^ from purified subunits to investigate assembly, disassembly and regulation of Atg1^PC^ as well as (2) reconstituting Atg9^core^ in proteoliposomes to biochemically dissect interactions of both components *in vitro* and *in vivo*. Our results led to a comprehensive model for Atg1^PC^-function, revealing insights into the molecular basis to initiate autophagy. We found that Atg17 has membrane-tethering activity, which is inhibited by the regulatory Atg31–Atg29 subcomplex. On dephosphorylation of Atg13, the Atg1–Atg13 subcomplex engages Atg17^TC^ to assemble Atg1^PC^. This releases the steric inhibition of Atg17, allowing Atg9 to bind the central crescent of Atg17. Consequently, each of the two Atg17-subunits in Atg1^PC^ binds an Atg9-vesicle, thereby tethering these vesicles together (Fig. 7d).

The most interesting insight of our study concerns the molecular function of Atg1^PC^ and its subunits at the PAS. A previous study established that an average of three Atg9-vesicles, 30–60 nm in size, coalesce at the PAS to nucleate the phagophore^10^. Here we demonstrate that Atg1^PC^ is a membrane-tethering factor, which specifically recognizes Atg9-vesicles of high curvature. The principle membrane-tethering subunit of Atg1^PC^ is Atg17, which has been shown to form elongated, S-shaped dimers in solution^11^,^12^. We could show that Atg31–Atg29 occludes the Atg9-binding site in Atg17, preventing Atg9-vesicles from being recruited under non-induced conditions by constitutive Atg17^TC^. Molecular basis for this competition are at least overlapping binding sites of Atg31^160–196^ and cytoplasmic domains of Atg9 in Atg17. Atg31^160–196^ forms a four-helical bundle with Atg17 by directly contacting helices α1 and α4^12^. Atg17-helix α4 also harbours the Atg9-binding site, thus preventing recruitment of Atg9 when Atg31 occupies the Atg17-crescent. The interaction of Atg17 with Atg9–PLs was depleted by 70–80% when Atg31–Atg29 was added to preassembled Atg17–Atg9–PLs *in vitro* or when Atg31^160–196^ was added to cell lysates *in vivo*. This suggests that a direct inhibition of Atg17 by Atg31–Atg29 is a key determinant in regulating the recruitment of Atg9-vesicles to the PAS in response to starvation. Moreover, the minimal Atg17-binding region of Atg31 is a very potent inhibitor of Atg17. Adding increasing amounts of Atg31^160–196^ to cell lysates which resulted in concentrations well above the estimated total concentration of Atg17 did, however, not result in full inhibition of the Atg17–Atg9 interaction. Residual binding might be mediated by Atg13, which has recently been found to bind Atg9 *in vitro* and *in vivo*^26^.

Assembly of Atg1^PC^ from its Atg17^TC^ and Atg1–Atg13-subcomplexes restores binding of Atg17 to Atg9 and promotes tethering of Atg9–PLs *in vitro*. The direct contact between both subcomplexes is mediated by Atg13, which directly interacts with Atg17 (refs 15, 39). This implies that the activation of Atg17 requires at least Atg13. In agreement with this notion, we found that not only the interaction between Atg17 and Atg9 is significantly impaired in *atg13*Δ cells, but also targeting of Atg9 to the PAS. Moreover, the phosphomimicking mutant Atg13^S428,429D^ does not form a pentameric complex with Atg17^TC^, thereby preventing Atg17 from interacting with and tethering of Atg9–PLs. A recent structural study identified the Atg13-binding site in Atg17, comprising helices α3 and α4 and being in close proximity to Atg31 (ref. 23). Moreover, the Atg31–Atg29 complex was suggested to be flexibly connected to Atg17 (ref. 12), thereby potentially adopting alternative conformations. Altogether with our observation that Atg13 activates Atg17^TC^ for Atg9-binding *in vitro* and *in vivo*, it appears plausible that Atg13 induces a conformational rearrangement in Atg17^TC^, such that Atg31–Atg29 move away from the central crescent to expose the Atg9-binding site of Atg17. This model is supported by our observation that although Atg13 did not directly interact with Atg9^core^, because Atg9^core^ lacks the previously identified Atg13-binding site^26^, Atg13 restores binding of Atg17^TC^ to Atg9–PLs to promote tethering of Atg9-vesicles. We therefore propose a model in which Atg13 exerts two different activities. First, Atg13 cooperates with Atg17 in recruiting Atg9-vesicles to the PAS by direct recognition of Atg9. Furthermore, Atg13 activates Atg17^TC^ such that fully assembled Atg1^PC^ binds and tethers Atg9-vesicles through its scaffolding subunit Atg17 (Fig. 7d).

Interestingly, we observed a significant difference in Atg1–Atg13-mediated activation of monomeric or dimeric Atg17^TC^. Even though Atg17^monoTC^ assembled together with Atg1–Atg13 into stable Atg1^monoPC^ *in vitro*, binding of Atg17 to Atg9 was not restored. Consistently, the low residual binding activity of Atg17^monoTC^ and Atg1^monoPC^ for Atg9 *in vitro* correlates with a lack of Atg9-recuitment to the PAS in Atg17^mono^-expressing cells. Atg17^mono^ was, however, also not recruited to the PAS on starvation, arguing that dimerization of Atg17 is not only important for regulating Atg17 activity, but also for its recruitment to the PAS.

Another interesting aspect of our study regards the membrane-binding activity of Atg1 and Atg13. Although the recombinantly expressed MIT-domain of Atg1 has been found to recognize membranes previously^12^, it was not clear how this affects Atg1^PC^. According to this study, we showed that full-length Atg1 also binds highly curved membranes. Moreover, we found that Atg1 possesses specificity for PI, thereby targeting Atg1^PC^ to PI-containing membranes with high curvature. While the MIT-domain of Atg1 has no structural similarity to canonical membrane binding domains^23^, a recent study showed that homologous MIT-domains in Vps4 and SNX15a bind PI and PI-phosphates as well^40^. Whether MIT-domains represent a new class of PI-specific membrane binding modules remains to be addressed by future studies. In contrast to our findings that Atg17 and Atg9 are not sensing membrane curvature, both proteins localize to the highly bent membrane edges of the phagophore^41^. Given that Atg1 and Atg13 sense curvature and that the Atg1–Atg13 subcomplex interacts with Atg17 at the PAS, it is tempting to speculate that Atg1–Atg13 restricts the localization of Atg17 and consequently that of Atg9 at the phagophore. This implies, on the other hand, that Atg1^PC^ might not only tether vesicles for phagophore nucleation, but also for phagophore expansion.

## Methods

### Reagents

Synthetic lipids 1-palmitoyl-2-oleoyl-sn-glycerol-3-phosphocholine (POPC), 1-palmitoyl-2-oleoyl-sn-glycerol-3-phosphoethanolamine (POPE), 1-palmitoyl-2-oleoyl-sn-glycerol-3-phosphoserine (POPS), 1-palmitoyl-2-oleoyl-sn-glycerol-3-phosphoinositol (POPI), cholesterol, 1,2-dioleoyl-*sn*-glycero-3-phospho-1′-myo-inositol-3′-phosphate (PI(3)-phosphate) as well as yeast polar lipid (YPL) extracts were purchased from Avanti Polar Lipids. The detergent *n*-Dodecyl-*N,N*-Dimethylamine-*N*-Oxide (LDAO) was from Anatrace.

The following antibodies were used in this study: αPep12p (Invitrogen, Catalog No: 710037, 1:1,000 dilution), αPgk1p (Invitrogen, Catalog No: 459250, 1:10,000 dilution), αHA (Santa Cruz, sc-7392, 1:200 dilution,), and αMyc (Santa Cruz, sc-789 and sc-40, 1:1,000 dilution), and αGFP antibodies (Roche, Catalog No: 11814460001).

### Yeast strains and growth conditions

Derivatives of yeast strain *Saccharomyces cerevisiae* BY4741 (Euroscarf) used for this study, are listed in Supplementary Table 2. Genomic gene deletion and tagging was performed as described^42^. Atg9-variants were ectopically expressed in *atg9*Δ cells. Therefore, the coding sequences of the respective Atg9-variants were sub-cloned into the pTL58 vector (pAtg9::Leu2) and transformed in *atg17*^*myc*^*atg9*Δ and *pho8*Δ*60atg9*Δ strains. To adjust the expression levels of Atg9 to that of its variants, the Pma1-promoter in pTL58-Atg9 was exchanged against the endogenous Atg9-promoter. To investigate whether Atg9^core^ is able to interact with Atg17 *in vivo*, pRS316-Atg9^core^-3xHA (pPma1::URA3) was transformed into *atg17*^*GFP*^*atg9*Δ cells. To analyse the regulation of Atg17 by Atg1 and Atg13 *in vivo*, pTL58-Atg9–3xHA (under control of the Atg9-promoter, pAtg9::Leu2) was transformed into *atg17*^myc9^*atg9*Δ*atg1*Δ and *atg17*^myc9^*atg9*Δ*atg13*Δ cells.

Co-localization of Atg17-variants and Atg8 was analysed by transforming plasmids pTL58-Atg17(variants)-mCherry (pPma1::Leu2)) in *atg8-GFP*Δ*atg11Δatg17Δ* strains. To study the co-localization of Atg17 and Atg9, pTL58-Atg17-2 × GFP (under control of its native promoter, pAtg17::Leu2) and pRS316-Atg9-2xmCherry (under control of its native promoter, pAtg9::URA3) were co-transformed into *atg17*Δ*atg9*Δ*, atg17*Δ*atg9*Δ*atg1*Δ, *and atg17*Δ*atg9*Δ*atg13*Δ cells.

Cells were grown in synthetic media (0.67% yeast nitrogen base, 0.5% ammonium sulphate, 2% glucose, amino acids) at 30 °C to mid-log phase. For co-immunoprecipitation experiments and Pho8Δ60 assays, autophagy was induced by starvation, cells were collected by centrifugation, washed twice and resuspended in synthetic medium without amino acids (0.17% yeast nitrogen base, 2% glucose). For fluorescence imaging, autophagy was induced by addition of rapamycin (0.2 μg ml^−1^, Sigma) for 1 h.

### Preparation of yeast cell extracts and immunoprecipitation

Yeast cells corresponding to 50 OD_600_ (optical density at 600 nm) were collected by centrifugation (3 min, 1,500*g*), resuspended in ice-cold lysis buffer (25 mM Tris-HCl pH 7.2, 150 mM NaCl, 0.2% NP-40 supplemented with protease inhibitor cocktail (Sigma) and 3 mM PMSF) and lysed by vortexing with acid washed glass beads. Lysates were cleared by centrifugation (20 min, 17,000*g*).

Immunoprecipitation was performed by incubating the cell lysates with an α-myc antibody coupled to magnetic protein A beads (raised from murine B-cell-hybridomas) or GFP-Trap-M beads (Chromoteck) for 2h at 4 °C. For competition assays, different amounts of recombinant, purified GST-Atg31^160–196^ were added to lysate. For GST pull-down assay, 10 μg of GST or GST-Atg31^160–196^ was incubated with cell lysate, prepared as described above. Lysates were incubated with Glutathione Sepharose 4B beads, extensively washed with lysing-buffer containing an additional 100 mM NaCl and resuspended in SDS-sample buffer. Samples were separated on NuPage Bis-Tris gels (Life Technologies) prior to immunoblotting. Corresponding uncropped blots are shown in Supplementary Fig. 7.

### Subcellular fraction assay

Yeast cells were grown to mid-logarithmic phase (OD_600_ of 0.8–1.0) and autophagy was induced by treatment with 0.2 μg ml^−1^ rapamycin for 1 h at 30 °C. Cells corresponding to 500 OD_600_ were collected by centrifugation (3 min, 1,500*g*) and resuspended in 50-ml buffer containing 100 mM Tris-H_2_SO_4_, pH 9.4 and 10 mM DTT. After incubation at 30 °C for 10 min, cells were spheroblasted by resuspending them in 50 ml synthetic medium containing 20 mM Tris-HCl pH 7.5 and 1.0 M sorbitol supplemented with 2.5 μg per OD_600_ zymolyase-100T (Biomol), and incubation for 30 min at 30 °C. Spheroblasts were washed twice in 50 ml synthetic medium containing 1.0 M sorbitol and lysed in 5-ml ice-cold hypoosmotic buffer (50 mM HEPES-KOH, pH 7.6, 200 mM sorbitol, 1 mM EDTA) containing complete protease inhibitors (Sigma) and 2 mM PMSF using 50 strokes in a Dounce homogenizer. The cell lysate was centrifuged sequentially at 300*g* for 5 min and 13,000*g* for 10 min and 1.9 ml of the supernatant was loaded on top of a 22–60% sucrose step gradient. The gradient was prepared by overlaying 0.5 ml 60% sucrose with 1.0 ml 37%, 1.5 ml 34%, 2 ml 32%, 2.0 ml 29%, 1 ml 27%, and 1.5 ml 22% sucrose solution. After ultra-centrifugation at 170,000 g for 17–18 h using a SW40Ti rotor (Beckman Coulter), 0.8 ml fractions were collected from top to bottom and analysed by western blotting using αPep12p (Invitrogen), αPgk1p (Invitrogen), αHA, and αMyc (Santa Cruz) antibodies.

### Pho8Δ60 assay

Autophagic activity was assessed as described^43^. Briefly, yeast cells corresponding to 4 OD_600_ were collected by centrifugation (3 min, 1,500*g*) and resuspended in ice-cold assay buffer (250 mM Tris-HCl, pH 9.0, 10 mM MgSO_4_ and 10 μM ZnSO_4_). Cells were lysed by vortexing with acid washed glass beads and cleared by centrifugation (20 min, 17,000*g*). 1-naphthyl-phosphate was added to final concentration of 5 mM and the enzymatic reaction was stopped after 20 min incubation at 30 °C by adding one volume of stopping buffer (2 M Glycin-NaOH, pH 11.0). The enzymatic conversion of 1-naphthyl-phosphate by alkaline phosphatases was determined by measuring the fluorescence with a TECAN infinite M1000Pro using an excitation wavelength of 345 nm and detecting emission at 472 nm. The fluorescence signal was corrected for the total protein content in the respective sample as determined using the Pierce BCA protein assay kit (Thermo Scientific).

### Recombinant expression and purification of the Atg1^PC^-subunits

The open reading frames encoding Atg1, Atg13, Atg17, Atg17^1–354^, Atg17^235–417^, Atg17^235–354^, Atg17^354–417^, Atg29, Atg31^160–196^ and Atg31 were amplified from yeast *S. cerevisiae* BY4741 (Euroscarf) genomic DNA and cloned into pCoofy-vectors with N- or C-terminal affinity-tags for expression in insect cells (Atg1) or *Escherichia coli* (all others) by homologous recombination^44^. Oligonucleotides used are shown in Supplementary Table 3. Atg17 and the Atg17-Atg31–Atg29 complex as well as its monomeric variant Atg17^1–354^-Atg31–Atg29 were (co)expressed from the polycystronic vector pST39 (ref. 45) with a His_6_-tag fused to the N-terminus of Atg17 or a myc-tag fused to the C-terminus of Atg17.

Atg1 was expressed in High Five (BTI-TN-5B1–4) cells applying the titerless infected-cells preservation and scale-up (TIPS) method as described^46^. Cells were resuspended in lysis buffer (100 mM Tris-HCl, pH 7.4, 300 mM NaCl, 20 mM imidazole, 5% glycerol, 5 mM β-mercaptoethanol, protease inhibitor cocktail (Sigma), benzonase (Sigma), 10 U ml^−1^) supplemented with 1% Triton-X and disrupted by high pressure homogenization. All other proteins were expressed in *E.coli* Rosetta cells. Cultures were grown in LB-medium at 37 °C until they reached an OD_600_ of 1.0 and shifted to 18 °C. Expression was induced by adding Isopropyl-β-D-thiogalactopyranosid (0.1 mM). The cells were collected by centrifugation after 16 h and resuspended in lysis-buffer. Cells were disrupted by sonication. All proteins were purified by Ni-nitrilotriacetate (Ni-NTA) affinity chromatography, applying a linear imidazole gradient in running buffer (25 mM Tris-HCl pH 7.4, 300 mM NaCl, 20 mM or 1 M imidazole, 5% glycerol, 5 mM β-mercaptoethanol). For Atg1 purification, a running buffer supplemented with 0.1% Triton-X was used. The N- and C-terminal tags were cleaved by PreScission protease digest for 30 min at room temperature. Digested proteins were immediately subjected to anion-exchange chromatography (Atg13) equilibrated with 25 mM Tris-HCl pH 7.4, 125 mM NaCl, 5% glycerol, 2 mM DTT or size exclusion chromatography (SEC) on a HiLoad Superdex 200 prep grade column (GE Healthcare) equilibrated with 25 mM Tris HCl pH 7.4, 300 mM NaCl, 5% glycerol, 5 mM β-mercaptoethanol. Fractions containing the protein of interest were pooled, concentrated and further purified by Ni-NTA rechromatography. Aliquots of the purified proteins were flash frozen in liquid nitrogen and stored at −80 °C until use. The Atg1–Atg13 subcomplex or Atg1^PC^ and its monomeric variant were assembled from purified subunits by incubating respective mixtures at room temperature for 30 min followed by gelfitration using a Superose 6 column.

### Expression and purification of Atg9

The coding sequence of Atg9^core^ (Atg9^281–779^) was amplified from *S. cerevisiae* BY4741 and sub-cloned into a modified pET28a(+)vector (Novagen). Truncated constructs Atg9^ΔN^ (residues 316–779), Atg9^ΔcD^ (residues 316–423 and 508–779) and Atg9^ΔNΔcD^ were generated using the In-Fusion HD Cloning Kit (Clontech). Atg9^core^ and its variants were expressed in *E. coli* Rosetta cells as described above, but resuspended in buffer containing 25 mM Tris-HCl, pH 8.0 and 150 mM NaCl. After lysing the cells with a high-pressure microfluidizer, cell debris was removed by centrifugation (10 min, 24,000*g*). The supernatant was subjected to a second centrifugation step to separate the membrane fraction (1 h, 150,000*g*). The membrane fraction was resuspended in buffer containing 25 mM Tris-HCl pH 8.0, 150 mM NaCl, and 40 mM LDAO (critical micelle concentration=1 mM) and incubated for 1 h at 4 °C to solubilize the membrane proteins. Not solubilized material was removed by ultracentrifugation (1 h, 150,000*g*) and the supernatant was purified by Ni-NTA-chromatography as described above, with Atg9-buffer (25 mM Tris-HCl pH 8.0, 150 mM NaCl, 4 mM LDAO), supplemented with imidazole. Atg9-containing fractions were pooled, concentrated and subjected to SEC using a Superdex-200 10/300GL column (GE Healthcare) for further purification using Atg9-buffer. Purified proteins were stored at −80 °C until used as described above.

### Circular dichroism spectroscopy

The quality of Atg9^core^ preparations was assessed using CD spectroscopy. CD-spectra of detergent-solubilized Atg9^core^ (-variants) were recorded using a JASCO J-715 spectrophotometer, covering a 190–250 nm spectral range, at 4 °C and a path length of 0.1 mm. The protein concentration was determined by measuring the OD at 280 nm and the secondary structure content of Atg9 was estimated by fitting experimental data with standard curves using the contin-algorithm^47^, provided by the Jasco Standard Analysis Program.

### Liposome preparation and sedimentation

Liposome sedimentation assays were performed to investigate protein–membrane interactions in the absence of Atg9. Liposomes were prepared from mixtures of synthetic lipids containing 20 mol% cholesterol, 10 mol% POPE, 60 mol% POPC, 10 mol% POPS or from YPL-extracts. For liposomes containing 30 mol% POPI, 25 mol% POPI+2.5 mol% PI(3)-phosphate, or 40 mol% POPS, the amount of POPC was adjusted accordingly. A thin lipid film was produced in a glass vial by evaporating the chloroform of the respective lipid-mixtures under a nitrogen-stream and subsequent incubation under vacuum. Multilamellar vesicles (MLVs) were generated by hydrating the lipid film with hydration-buffer containing 25 mM Tris-HCl pH 8.0, 150 mM NaCl, adjusted to a total lipid concentration of 1 mg ml^−1^. Large unilamellar vesicles were produced by extruding the resulting MLV-suspension through filters with appropriate pore size as indicated. SUVs were prepared by sonicating the MLV-suspension on ice until the solution became clear followed by extrusion through 30-nm pore-size filters.

Liposome sedimentation assays were performed by incubating 100-μl large unilamellar vesicles or SUVs with the protein of interest (2 μM final concentration) for 30 min at room temperature. The liposomes were collected by centrifugation (30 min, 140 000 g, 4 °C) for subsequent analysis using SDS–PAGE.

### Reconstitution of Atg9 in liposomes and floatation assay

Atg9^core^ or its variants were reconstituted in liposomes using the rapid dilution approach. Liposomes were prepared from mixtures of synthetic lipids containing 20 mol% cholesterol, 10 mol% POPE, 60 mol% POPC and 10 mol% POPS, yielding a total lipid concentration of 1 mg ml^−1^. After rehydration of liposomes, lipids were resolubilized with LDAO yielding a final concentration of 6 mM LDAO before Atg9-variants were added. Atg9^core^-(variants) solubilized in 4 mM LDAO were added in a protein:lipid ratio of 1:200 and the mixture was 30-fold diluted with hydration buffer and incubated for 30 min at room temperature. Atg9 containing proteoliposomes (Atg9-PL) were collected by centrifugation (30 min, 150 000 g, 4 °C), resuspended in 25 mM HEPES pH 7.0, 100 mM NaCl (interaction buffer) and extruded using a filter pore size of 200 nm.

The orientation of Atg9 was assessed by incubating Atg9-PL with PreScission protease (50 U ml^−1^) at room temperature for 1 h. Proteolytical cleavage of the C-terminal His-tag resulted in a significant band shift of the corresponding protein on SDS–PAGE gels.

The interaction of Atg1^PC^, its subunits, or its subcomplexes with Atg9-PL was assessed by floatation experiments. Therefore, 250 μl of Atg9–PLs were mixed with an equivalent volume of 80% Nycodenz in interaction buffer containing the respective Atg1^PC^-subunits in a stoichiometric ratio of 1:1. A step gradient was generated by overlaying this mixture with 250 μl 30% Nycodenz and 100 μl interaction buffer. After centrifugation at 650,000*g* for 2 h (at 4 °C), the top 100 μl co-floated fraction contained accumulated Atg9–PLs (at the Nycodenz–buffer interface) and bound interaction partners. This fraction was subsequently analysed by SDS–PAGE and immunoblotting.

### Dynamic light scattering

The protein amount used for tethering assays was calculated according to the amount of Atg9–PLs with native orientation in the membrane (50% of total protein). For each assay, 200 μl Atg9–PLs were mixed with 100 μl protein of interest, yielding molar ratios of 1:1, diluted in 25 mM HEPES pH 7.0 and 100 mM NaCl buffer and incubated at room temperature for 15 min. The mixture was co-sonicated at 4 °C until the solution appeared clear (15 min). Dynamic light scattering was carried out at 4 °C on a DynaPro NanoStar Instrument (Wyatt) using 50-μl cuvettes and a total sample volume of 20 μl. Raw data were analysed by the DYNAMICS software package. The dispersity of the solution was assessed, and the average hydrodynamic radius (*R*_H_) was calculated.

### Multi-angle light scattering

Size exclusion chromatography–MALS (SEC–MALS) measurements were performed on a HP1100 HPLC system (Agilent) using a Superdex-200 Increase 10/300GL (GE-Healthcare, Atg1) or a Superose-6 10/300GL (GE-Healthcare, Atg1^PC^) column. The MALS signal was recorded on-line using a miniDAWN Treos MALS detector (Wyatt) and the corresponding protein concentration was determined with an Optilab t-Rex RI detector (Wyatt). Atg1 was injected at a concentration of 5 mg ml^−1^ and Atg1^PC^ at a concentration of 3.4 mg ml^−1^ in a volume of 50 μl and 100 μl, respectively. SEC–MALS data were analysed using the Astra software package (Wyatt) applying the Zimm light scattering model. The molar mass at the concentration peak apex (MW max, refractive index peak apex) and the weight-average molar mass of the respective peak (MW peak, ultraviolet peak borders selected by hand) were determined.

### Tandem mass spectrometry

Proteins for tandem mass spectrometry were used at a 1 μM concentration. Proteolytic digestion was performed over night with LysC at RT and subsequently with Trypsin for 2 h at 37 °C. For injection to the LTQ Orbitrap (Thermo Fisher) tandem mass spectrometer, the resulting peptides were separated by C18 reversed phase nanoscale liquid chromatography. Data were analysed using the MaxQuant software package^48^.

### Confocal microscopy

Images were acquired on a Zeiss LSM780 and a Leica TSC SP8 laser scanning confocal microscope with × 63/1.4 numerical aperture (NA) objectives using the Zeiss ZEN 2011 and LAS AF software packages, respectively. GFP and mCherry were excited with 488-nm and 561-nm laser light, respectively. Multi-tracking mode was used for image acquisition on the Zeiss LSM780 microscope. Pinholes were set for acquiring 1.5–2.0 μm optical slices. All images were analysed using ImageJ (Schneider *et al.*, 2012) and Zeiss ZEN 2011 software packages. For yeast cell imaging, 5 ml yeast cells were grown to log phase and treated with rapamycin for 1 h. Two hundred-microlitre samples were added to an observation chamber (Lab-Tek #1.0 Borosilicate), which had been coated with 1 mg ml^−1^ concanavalin A (Sigma).

### Cryo-EM analysis

Samples (5 μl co-sonicated Atg9–PLs) were applied to lacey-carbon-grids (Quantifoil), blotted and frozen in liquid ethane using a Vitrobot (FEI). Images were recorded on a CM120 FEG under low-dose conditions with a 4k × 4k CCD camera (TVIPS) and a defocus of −2 μm.

## Additional information

**How to cite this article:** Rao, Y. *et al.* The Atg1–kinase complex tethers Atg9-vesicles to initiate autophagy. *Nat. Commun.* 7:10338 doi: 10.1038/ncomms10338 (2016).

## Supplementary Material

Supplementary Information Supplementary Figures 1-7, Supplementary Tables 1-3 and Supplementary Reference
