# Supplementary material for: The Atg1–kinase complex tethers Atg9-vesicles to initiate autophagy
Source: Nat Commun. 2016 Jan 12;7:10338. doi: 10.1038/ncomms10338 (PMC4729957; doi:10.1038/ncomms10338)
Supplement: Supplementary Information — Supplementary Figures 1-7, Supplementary Tables 1-3 and Supplementary Reference [file ncomms10338-s1.pdf]

Supplementary Figures

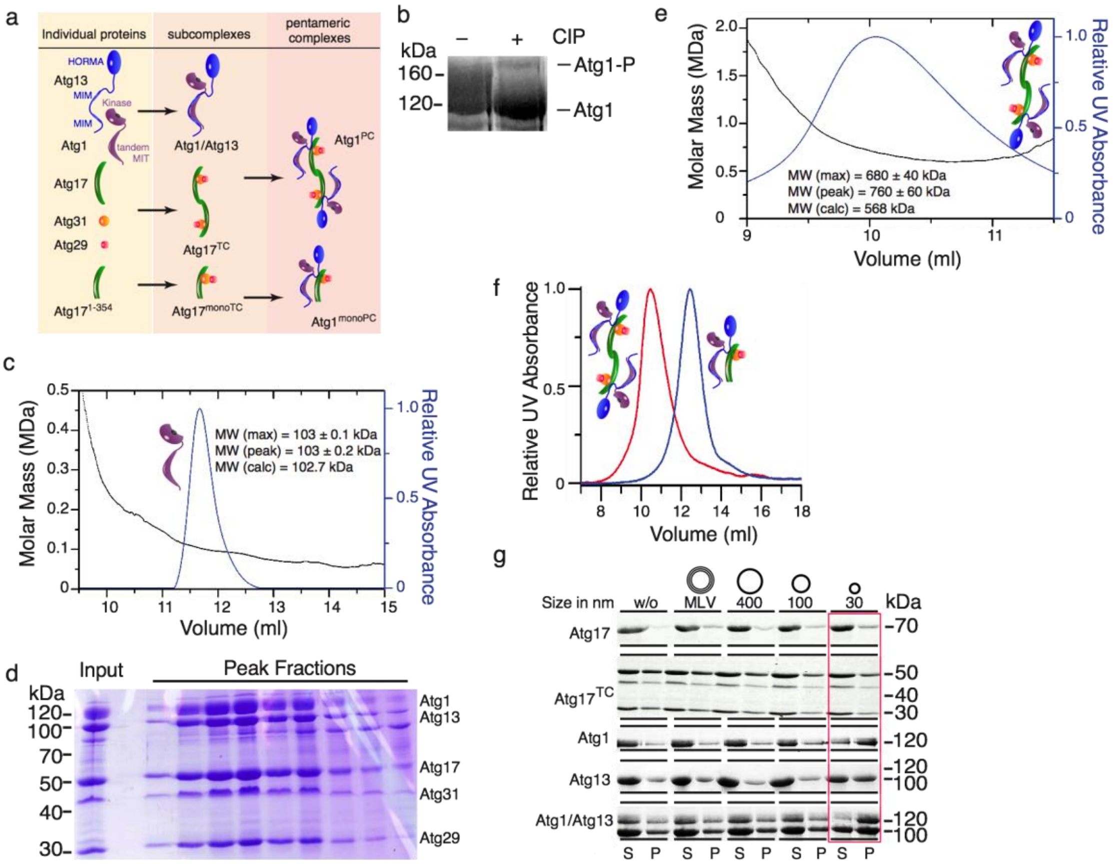

Supplementary Figure 1 Related to Figure 1. (**a**) The schematic drawing shows cartoons, representing the indicated subunits of the Atg1-kinase complex (individual proteins). Atg17 forms stable dimers in solution, however Atg17^{mono}, which corresponds to a C-terminal truncated variant, does not dimerize. The hierarchical association of the subunits is illustrated as follows: subcomplexes represent stable entities which are formed in vitro irrespective of their phosphorylation state. Atg17^{TC} refers to the Atg17-Atg31-Atg29 (dimeric) subcomplex and Atg17^{monoTC} to the Atg17$^{1\text{-}354}$-Atg31-Atg29 (monomeric) subcomplex. A pentameric complex assembles upon dephosphorylation of the complex subunits. Atg17 promotes the assembly of a dimeric pentameric complex (Atg1^{PC}) from Atg17^{TC} and Atg1-Atg13, whereas Atg17$^{1\text{-}354}$ forms a monomeric pentameric complex (Atg1^{monoPC}). **(b)** Recombinant Atg1-kinase was dephosphorylated by calf intestinal alkaline phosphatase (CIP). Electrophoretic mobility shift of the treated sample indicates that Atg1-kinase is partially phosphorylated (Atg1-P). **(c)** SEC-MALS profiles of Atg1 and corresponding absolute molecular weights in solution. The black curve shows the size distribution, determined by MALS, whereas the blue curve represents the SEC-elution profile. MW (max) = molecular weight determined from the peak-maximum of the SEC elution profile, MW (peak) = molecular weight average determined form the entire peak, MW (calc) = theoretical molecular weight determined form the amino acid sequence(s). **(d)** SDS-PAGE of peaks fractions from SEC of Atg1^{PC} as shown in **Figure 1b**. **(e)** SEC-MALS profiles of Atg1^{PC} and corresponding absolute molecular weights in solution. **(f)** SEC elution profiles of Atg1^{PC} (red) compared to that of Atg1^{monoPC} (blue). Cartoons represent subunits and complexes as indicated. **(g)** Liposome sedimentation assay of Atg1^{PC}-subunits as indicated with YPL- MLVs or YPL-LUVs of defined sizes to analyze curvature dependent membrane interactions. S = supernatant, P = pellet. The red box highlights best binding conditions.

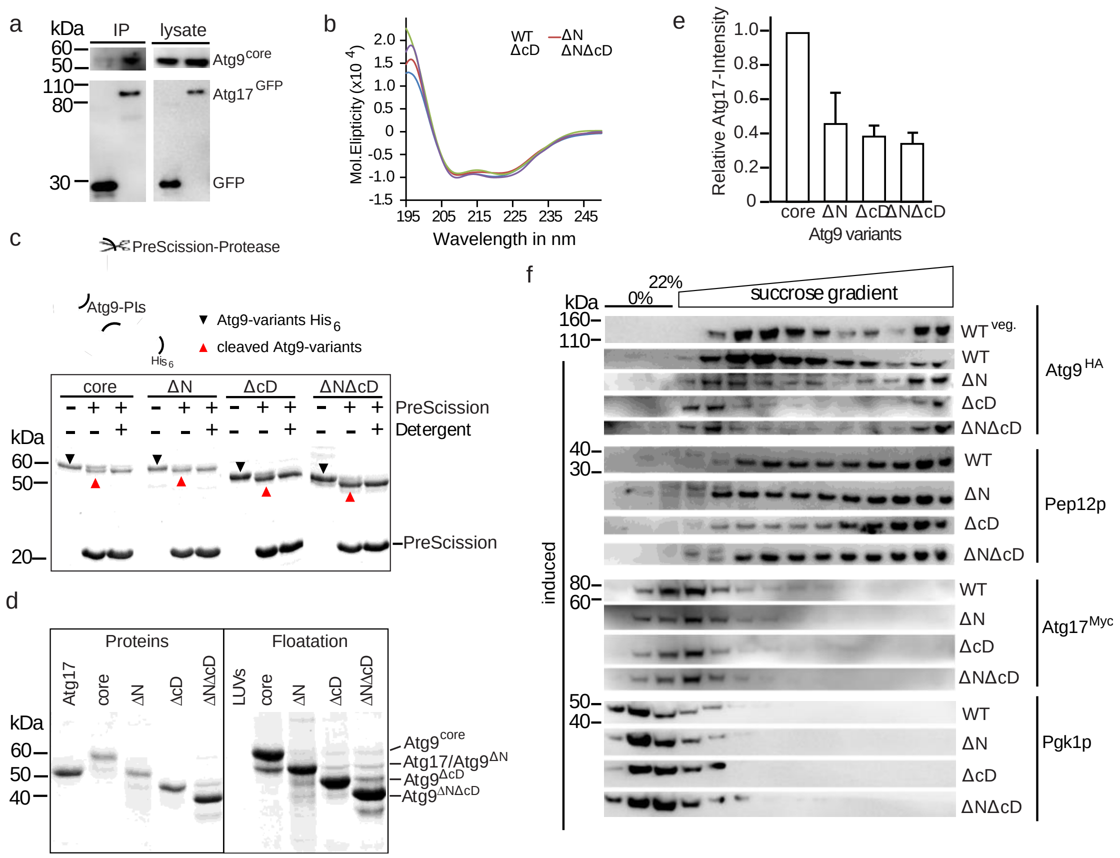

Supplementary Figure 2 Related to Figure 2. (**a**) Immunoprecipitation of Atg17^{GFP} and GFP from lysates of *ATG9*Δ, and *ATG9*$^{core, HA}$ (*ATG9*$^{281\text{-}779}$), after 2 h of starvation. Atg17^{GFP} and co-immunoprecipitated (co-IP) Atg9$^{core, HA}$ were detected by α-GFP and α-HA immunoblots as indicated. An efficient binding of Atg17 and Atg9^{core} is observed. (**b**) Circular dichroism spectroscopy of Atg9-variants in buffer containing LDAO. The recorded elipticity (represented by circles) was fitted using the contin-algorithm to determine the secondary structure content of Atg9 [1]. Atg9-variants are indicated by different colors. (**c**) Analysis of Atg9-orientation in Atg9-PL. Atg9 was expressed with a cleavable C-terminal His$_6$-tag. Upon addition of PreScission protease, all exposed (i.e. extraluminal) His-tags were cleaved. Intraluminal C-termini are protected from cleavage. Atg9-variants (black arrowhead) and Atg9-variants-His$_6$ (red arrowhead) were separated by SDS-PAGE. Exposed C-termini correspond to Atg9 with native topology, whereas the protease protected fraction of Atg9 was inversely incorporated into vesicles with cytoplasmic domains, facing the lumen of liposomes. (**d**) SDS-PAGE of recombinant proteins (left) and the top (floating) fraction from floatation experiments (right) of Atg9-PL (containing Atg9^{core} or its variants as indicated) with Atg17. Similar floatation experiments using LUVs lacking Atg9^{core} were used as control. (**e**) Semi-quantitative analysis of immunoblots as shown in **Figure 2b**. The chart shows integrated band intensities of Atg17. The intensity of Atg17^{myc} from floatation experiments with Atg9-PLs were set to one and used for normalization. Mean values ± SD of N=3 independent experiments. (**f**) Subcellular fraction of lysates prepared from HA-tagged Atg9^{WT} (WT), Atg9^{ΔN} (ΔN), Atg9^{ΔcD} (ΔcD), and Atg9^{ΔNΔcD} (ΔNΔcD) expressing cells. The supernatant of 13,000 g centrifuged cell lysates was applied to a sucrose-gradient and ultra-centrifuged. Fractions were taken from top (0% sucrose) to bottom (60% sucrose) and analyzed by SDS-PAGE and immuno-blotting. Atg9 and Atg17 were immuno-detected using α-HA and α-myc antibodies, respectively. Pep12p and Pgk1p were detected using antibodies raised against peptides of the corresponding proteins. WT^{veg} shows Atg9 distribution of lysates in non-induced cells. All other lysates have been prepared after induction of autophagy by rapamycin-treatment. The distribution of Atg9^{ΔN}, Atg9^{ΔcD}, and Atg9^{ΔNΔcD}

(autophagy induced) is similar to that of Atg9^{WT} (non-induced). The shift of Atg9$^{\Delta cD}$- and Atg9$^{\Delta N\Delta cD}$-distributions to lower densities is due to the considerable loss in protein mass due to corresponding truncations.

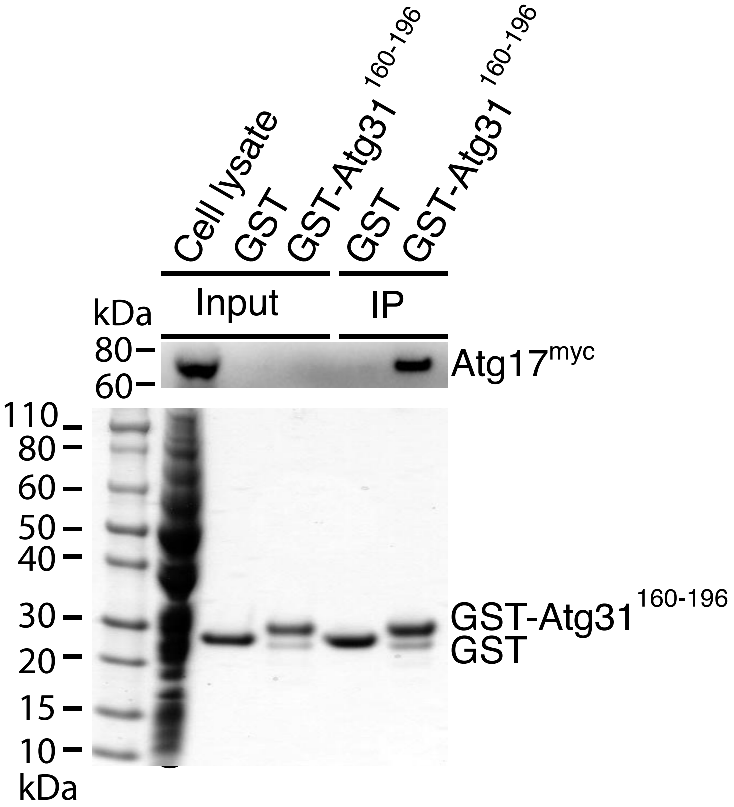

Supplementary Figure 3 Related to Figure 3. The interaction between Atg17^{myc} and GST-Atg31$^{160\text{-}196}$ from lysates was performed by GST pull-down assay. Yeast cells expressing Atg17^{myc} were starved for two hours and cell lysates were prepared. 10 µg recombinant, purified GST-Atg31$^{160\text{-}196}$ or GST (control) were coupled to Glutathione Sepharose 4B beads, incubated with cell lysates, extensively washed and mixed with SDS loading buffer. Samples were analyzed by SDS-PAGE and western-blotting using αmyc antibody. Coomassie stained gel shows loading controls of GST and GST-Atg31$^{160\text{-}196}$.

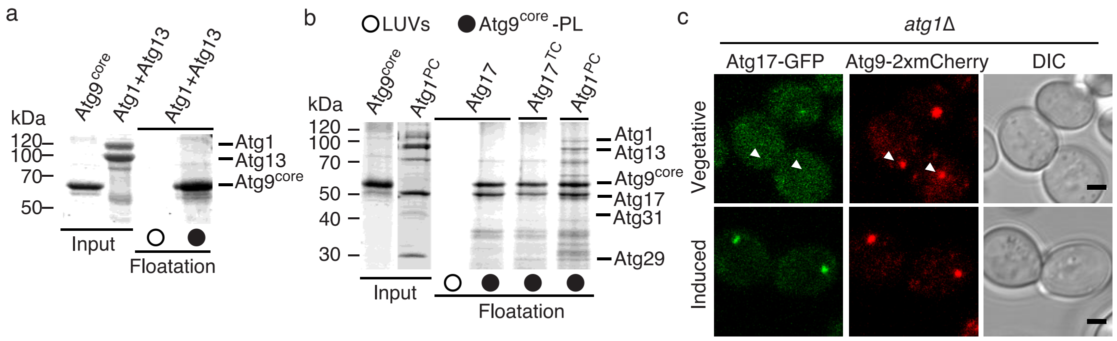

Supplementary Figure 4 Related to Figure 4. (**a**) SDS-PAGE of floating fractions from co-floatation experiments of Atg9-PL and LUVs prepared from synthetic lipid mixtures lacking phosphatidylinsositol with Atg1 and Atg13. Both Atg1 and Atg13 do not interact with Atg9-PLs. **(b)** SDS-PAGE of the top (floating) fraction from co-floatation experiments of Atg9-PL or LUVs with Atg17, Atg17^{TC} and Atg1^{PC} as shown in **Figure 4b**. (**c**) Co-localization of Atg9 and Atg17 in *atg1*Δ cells under vegetative and rapamycin-induced conditions. Cells co-expressing Atg9-2xmCherry and Atg17-GFP were grown to log phase and treated with rapamycin for 1h before imaging. Arrowheads indicates non-colocalizing Atg9-puncta. Scale bar = 2 μm.

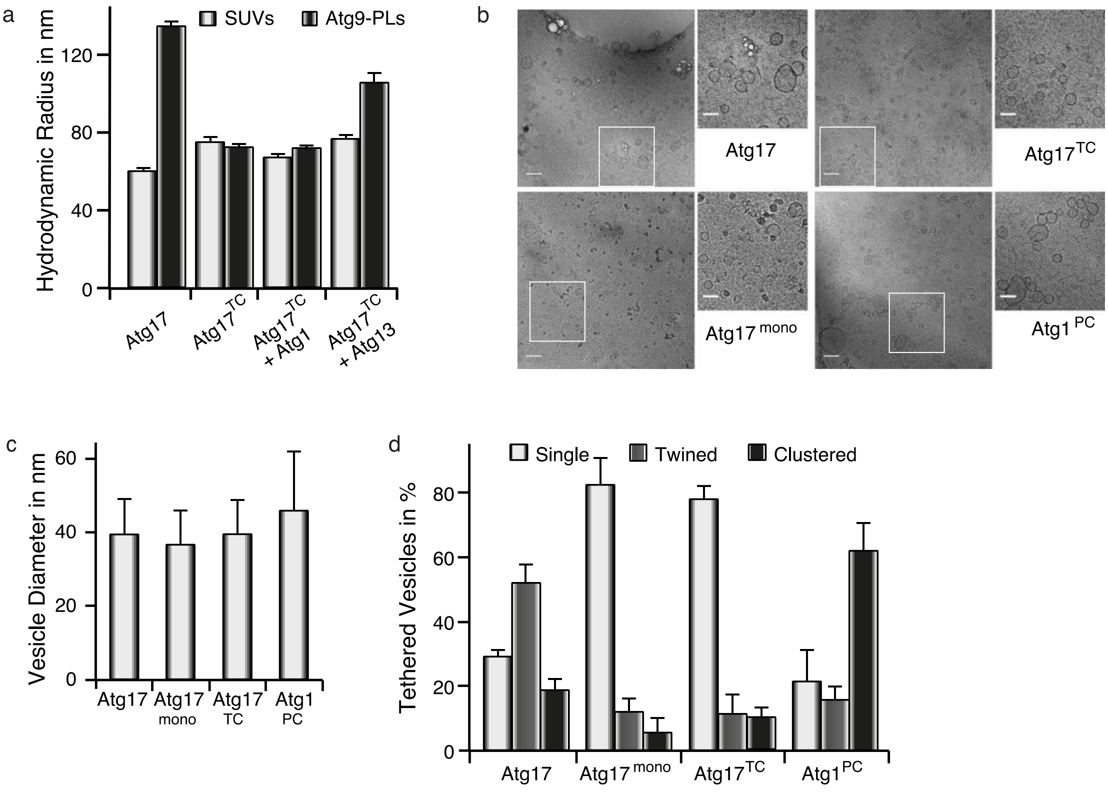

Supplementary Figure 5 Related to Figure 6. **(a)** Hydrodynamic radii of SUV (white bar) and Atg9-PLs (black bar) obtained from light scattering experiments with Atg17, Atg17^{TC}, Atg17^{TC} + Atg1, and Atg17^{TC} + Atg13 are shown. Respective Atg1^{PC}-subunits were co-sonicated with vesicles to prevent Atg9-mediated clustering. Atg13 increased the hydrodynamic radius and polydispersity of Atg9-PLs significantly, indicating that the inhibition in Atg17^{TC} for Atg9-binding is partially released, leading to inefficient tethering of Atg9-PLs. **(b)** Cyro-electron micrographs of Atg9-PLs, co-sonicated with Atg17, Atg17^{TC}, Atg17^{mono}, or Atg1^{PC}. Insets correspond to images shown in **Figure 6d**. Scale bar, 100 nm and 50 nm for overview and insets, respectively. (**c**) Quantification of the diameter of vesicles from cyro-electron micrographs of indicated samples. Mean ± SD from 21 vesicles, randomly selected from micrographs of two grids corresponding to two independent experiments are shown. (**d**) Quantification of vesicle tethering. Vesicles from randomly selected areas of micrographs from three grids corresponding to three independent experiments were analyzed. Two vesicles with significant contacting area were counted as twined, more vesicles with significant contacting areas were counted as clustered, and those without contact to other vesicles as single. Data are based on >180 vesicles for each condition.

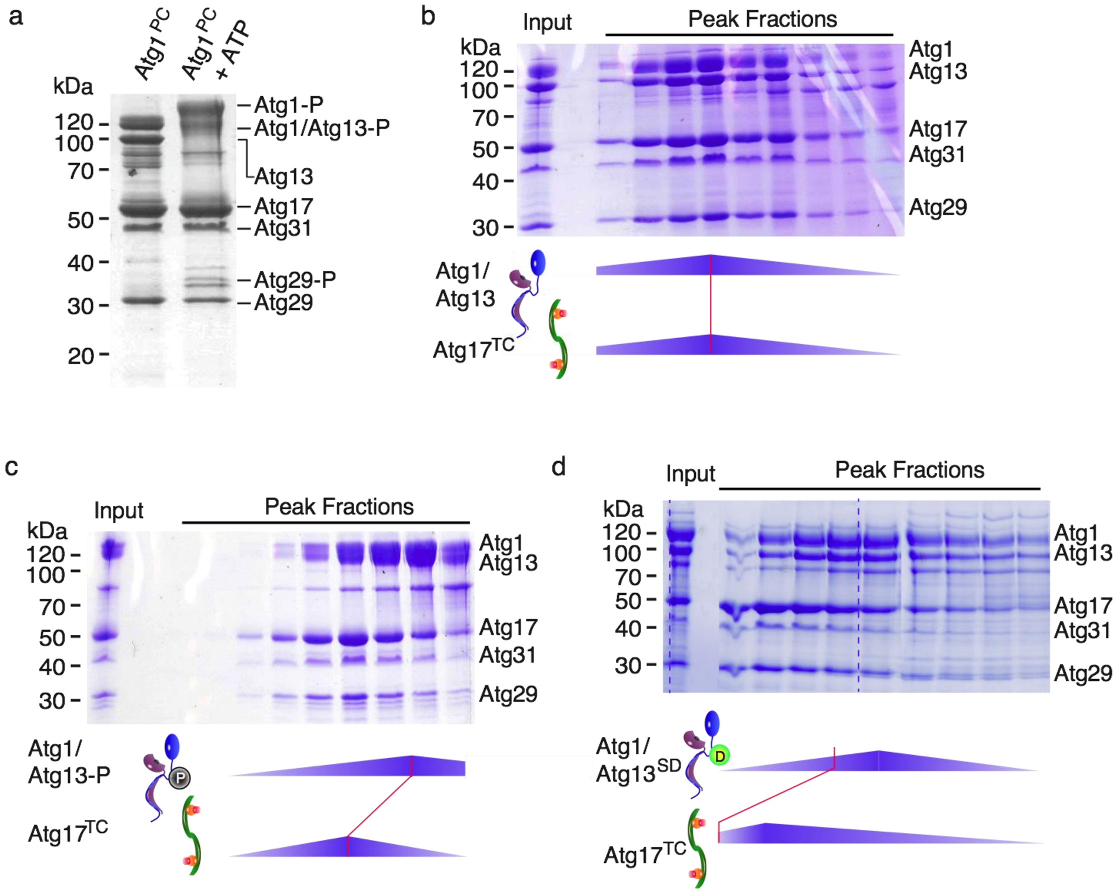

Supplementary Figure 6 Related to Figure 7. (**a**) SDS-PAGE of Atg1^{PC} in the presence and absence of 1 mM ATP. Shifts in electrophoretic mobility correspond to phosphorylation of subunits by Atg1-kinase. **(b-d)** SDS-PAGE of peak fractions from SEC experiments of Atg1^{PC} without (**b**) and with addition of 1 mM ATP (**c**), and Atg17^{TC} + Atg1-Atg13^{S428DS429D} (**d**). Fractions correspond to elution profiles shown in **Figure 7a**. Both addition of ATP and substitution of Atg13 by its phosphor-mimetic Atg13^{SD} prevent formation of Atg1^{PC} as demonstrated by distinct band-intensity maxima of Atg17^{TC} and Atg1-Atg13 subcomplexes.

Figure 1c

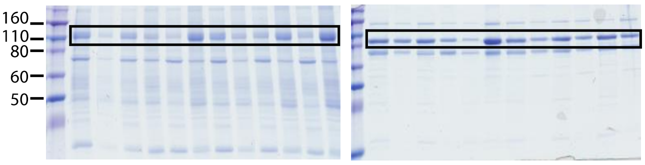

Supplementary Figure 1g

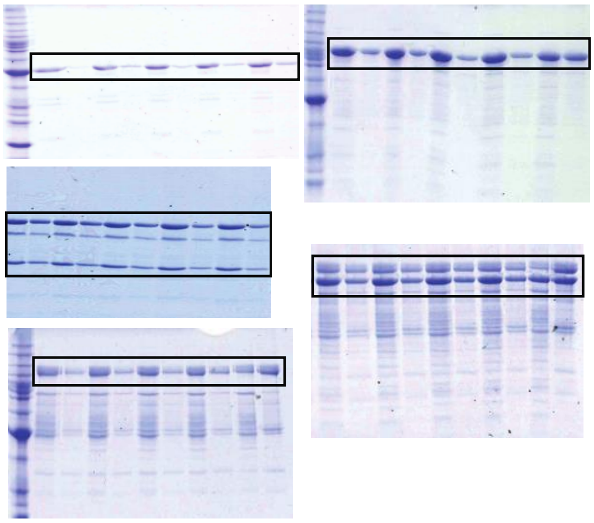

Figure 2c

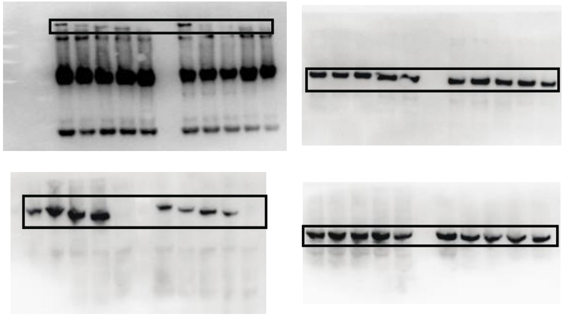

Supplementary Figure 2a

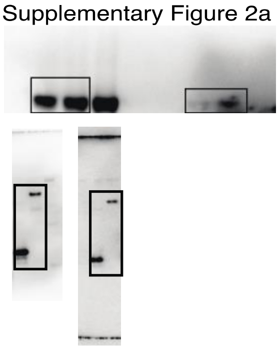

Figure 2b

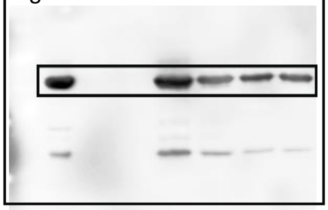

Supplementary figure 2f

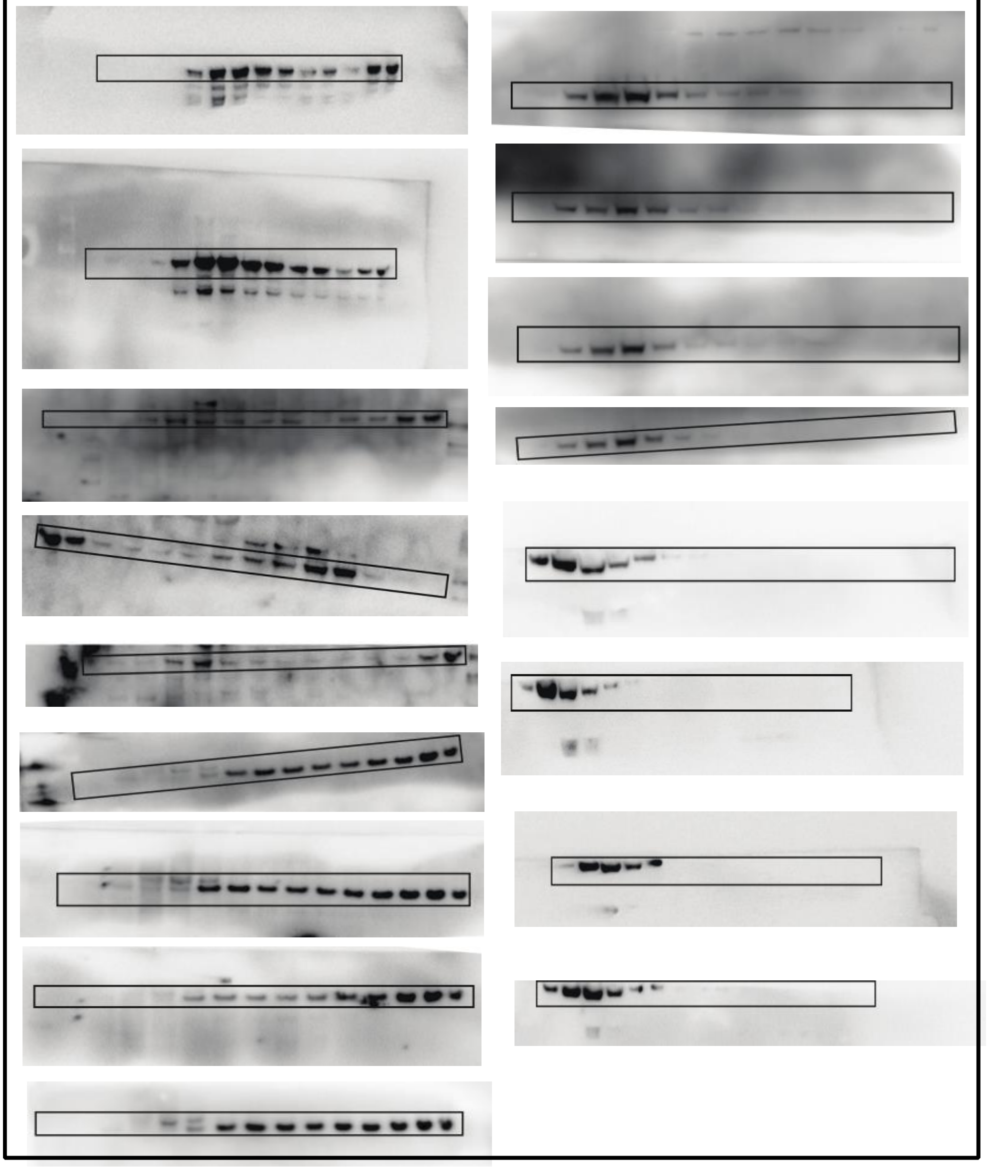

Figure 3c

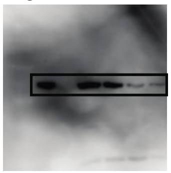

Supplementary 3

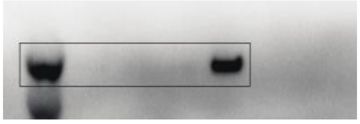

Figure 4b

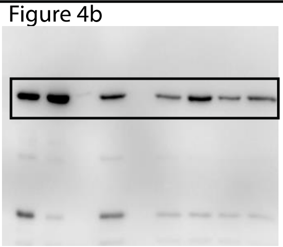

Figure 3e

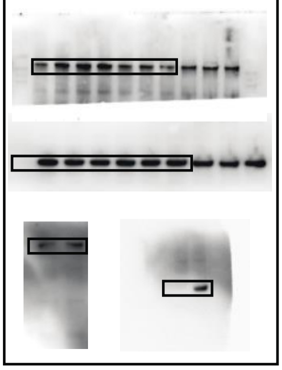

Figure 4d

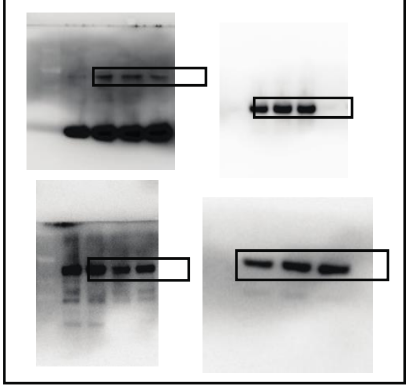

Figure 5a

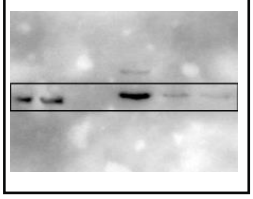

Figure 7b

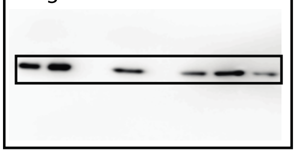

Supplementary Figure 7 Related to Figures 1, 2, 3, 4, 5, 7 and Figures S1 and S2. Original immunoblots and SDS-PAGE-gels, which have been cropped in main and supplemental figures, are shown. Frames indicate shown bands in indicated main and supplemental figures.

Supplementary Table 1: Atg1 kinase phosphorylates Atg1^{PC} subunits in vitro.

Atg1

Positions of phosphorylations	Peptide sequence	w/o ATP (log(intensity))	+ 1 mM ATP (log(intensity))
T226	FLPNTSLAE**T**LCGSPLYMAPEILNYQK	7.8	8.3
S351 or S356	GIVE**S**NMFV**S**EYLSK	0	7.9
S351 and S356	GIVE**S**NMFV**S**EYLSK	0	8.3
S533	ALGIA**S**TR	0	7.7

Atg13

Positions of phosphorylations	Peptide sequence	w/o ATP (log(intensity))	+ 1 mM ATP (log(intensity))
S346	SL**S**LSPCTR	0	8.7
S428 or S429	YS**SS**FGNIR	0	8.5
S428 and S429	YS**SS**FGNIR	0	6.6
S496	KTSGNNPPNINISD**S**LIR	0	7.6
(S535 and S543) or (S533 and S543) or (S533 and S541)	GRSD**S**H**S**PLPSI**S**P**S**MHYGSLNSR	0	8.5
S643 or S644	FK**SS**ISPR	0	8.5
(S649 and S654) or (S649 and S656) or (S652 and S656) or (S655 and S656)	**S**ID**S**I**SSS**FIK	0	8.7

Atg29

Positions of phosphorylations	Peptide sequence	w/o ATP (log(intensity))	+ 1 mM ATP (log(intensity))
S5 or T8	MIMN**S**TN**T**VVYIK	5.6	6.6
S106	YSNDQVNEGM**S**DLIHK	0	8.8
S125	YTPTLQNDNLLNVSASPLTTER	0	8.6
S136 or T144 or S154	QD**S**EEVETEV**T**NEALQHLQT**S**K	0	8.7
S201	**S**ALEEALMDR	0	7.6

Supplementary Figure 1 Related to Figure 7. Atg1 kinase phosphorylates Atg1^{PC} subunits in vitro. SEC purified Atg1^{PC} was incubated with or without 1 mM ATP for 30 min at 30 °C and subsequently digested in solution by LysC and Trypsin. The resulting peptides were analyzed using C18 reversed phase nanoscale liquid chromatography coupled tandem mass spectrometry. Some peptides were found to be phosphorylated at different and/or multiple positions as indicated. The table lists the previously identified regulatory sites in Atg1 and Atg13 that were also phosphorylated in our analysis (except for Atg13 in which S346 was phosphorylated instead of S348). Serine residues highlighted in green were mutated to aspartate to generate the phosphomimetic Atg13^{SD}-variant. Furthermore, all phosphorylation sites detected in Atg29 are listed. Atg29 phosphorylated at S5 or T8 in the absence of ATP represents a minor fraction of the preparation, because no mass corresponding to phosphorylated Atg29 was detected by electron spray ionization (ESI)-MS and the electrophoretic mobility of Atg29 on SDS-PAGE gels did not change after phosphatase-treatment. The listed intensities are average values from four independent experiments.

Supplementary Table 2: Stains used in this study.

Name	Genotype
atg17-myc$_9$ atg9Δ	atg17Δ::atg17myc$_9$::hphNT1 atg9Δ::kanMX
atg17-GFP atg9Δ	atg17-GFP::hphNT1 atg9Δ::kanMX
atg17-myc$_9$ atg9Δ atg1Δ	atg17Δ::atg17myc$_9$::hphNT1 atg9Δ::kanMX atg1Δ:: natNT2
atg17-myc$_9$ atg9Δ atg13Δ	atg17Δ::atg17myc$_9$::hphNT1 atg9Δ::kanMX atg13Δ:: natNT2
pho8Δ60 atg9Δ	pho8Δ::pho8Δ60::natNT2 atg9Δ::kanMX
pho8Δ60 atg17Δ	pho8Δ::pho8Δ60::natNT2 atg17Δ::hphNT1
pho8Δ60 atg17-(1-354)	pho8Δ::pho8Δ60::natNT2 atg17-(1-354)::hphNT1
Atg8-GFP atg17Δ atg11Δ	Atg8-GFP::natNT2 atg17Δ::hphNT1 atg11Δ::kanMX
atg17Δ atg9Δ	atg17Δ::hphNT1 atg9Δ::kanMX
atg17Δ atg9Δ atg1Δ	atg17Δ::hphNT1 atg9Δ::kanMX atg1Δ::natNT2
atg17Δ atg9Δ atg13Δ	atg17Δ::hphNT1 atg9Δ::kanMX atg13Δ::natNT2

Supplementary Table 3: Oligonucleotides used in this study.

Protein	Primer
**Atg9-(281-779) for**	TCTACCATGGGCAGCGTGAAAGAGCGGGCTCTGTGG
**Atg9-(281-779) rev**	TCTAGCTAGCAGCGTGAAAGAGCGGGCTCTGTGG
**atg17 for**	CAGACTCCGGAAATAATTTTGTTTAACTTTAAGAAGGAGATATACATATGGGCAGCAGCcatcaccatcaccatcacATGAACGAAGCAGATGTTACAAAATTTGTTAATAATGCC
**atg17 rev**	GTCTGacgcgtCTAAGGATTCTTCACGTTGTAATTTAAAGTGTACAGGG
**atg29 for**	CAGACgaattcAATAATTTTGTTTAACTTTAAGAAGGAGATATACATATGATTATGAATAGTACAAACACAGTTGTATATATC
**atg29 rev**	GTCTGaagcttTCAGAATTGCAATCTGTCCATTAGCGC
**atg31 for**	CAGACtctagaAATAATTTTGTTTAACTTTAAGAAGGAGATATACATATGAATGTTACAGTTACTGTTTATGATAAAAATGTC
**atg31 rev**	GTCTGggatccTCATACGGAATTGGAGAGCATTTGTAATTGTTC
**atg1 for**	CactaaagggcggccATGGGAGACATTAAAAATAAAGATCACAC
**atg1 rev**	attgttaattaagagttaATTTTGGTGGTTCATCTTCTGCC
**atg13 for**	Tagggcccgggcgtc ATGGTTGCCGAAGAGGACATC
**atg13 rev**	GCGGTACCAAGCTTAACCTTCTTTAGAAAGGTTCATATCACTCATG
**atg13(S428 429D)**	GTTGGAAGCACCTCAAAGTATTCCGACGACTTTGGGA

**for**	ACATTCGTCGTC
**atg13(S428 429D) rev**	GACGACGAATGTTCCCAAAGTCGTCGGAATACTTTGAGGTGCTTCCAAC
**atg17(1-354) for**	CTAGAAGCAAAGAGGAGAAAGGATGAACAGAAACTGATTAGCGAAGAAGATCTGTAGGGCAAACAGAATGAAAACTATATTG
**atg17(1-354) rev**	CAATATAGTTTTCATTCTGTTTGCCCTACAGATCTTCTTCGCTAATCAGTTTCTGTTCATCCTTTCTCCTCTTTGCTTCTAG
**atg17(235-417) for**	aagttctgttccaggggcccggtggcagcagcggtagcggtagcagcggtGATGAACGTGAGGAGCTGTTTAAG
**atg17(235-417) rev**	GAACATCAGGTTAATGGCGTCTAAGGATTCTTCACGTTGTAATTTAAAG
**atg17(235-354) for**	aagttctgttccaggggcccggtggcagcagcggtagcggtagcagcggtGATGAACGTGAGGAGCTGTTTAAG
**atg17(235-354) rev**	GAACATCAGGTTAATGGCGTCTAATCCTTTCTCCTCTTTGCTTCTAGAAC
**atg17(354-417) for**	aagttctgttccaggggcccggtggcagcagcggtagcggtagcagcggtGATGTGGCAAACAGAATGAAAAC
**atg17(354-417) rev**	GAACATCAGGTTAATGGCGTCTAAGGATTCTTCACGTTGTAATTTAAAG
**Atg31-(160-196) for**	CCACCGGATCCATTTCACAATTTTGCGATCTGTCTCCTTTTCTTCG
**Atg31-(160-196) rev**	ATTCCTCGAGTCATACGGAATTGGAGAGCATTTGTAATTG
**Atg9-Δ(423-508) for**	AACGAGTCCGGATTTATTAAACAAAGT
**Atg9-Δ(423-508) rev**	AAATCCGGACTCGTTGGGCAAAGTTTGAAGTTCGTCATCCG
**Atg9-Δ(281-315) for**	AACGTAGAAAACCTCGATATATTTCTTCAAGATGTTTACAATTATTATCTGGGAAATGGGTTCTATTGCATCATAC
**Atg9-Δ(281-315) rev**	CGAGGTTTTCTACGTTTGCCCATTTCCACAGAGCCCGCTCTTTCACGCTCAACGTATTGATTCTATCTTCCGGGGTATTGTTTAAAACGG

Supplementary Reference

1. Provencher, S. W. & Glöckner, J. Estimation of globular protein secondary structure from circular dichroism. *Biochemistry* **20,** 33–37 (1981).
